# Advanced Targeted Curcumin Delivery Using Spatiotemporally Controlled Nanohybrid Polysaccharide-Based Hydrogel for Ulcerative Colitis Therapy

**DOI:** 10.3390/gels12060503

**Published:** 2026-06-05

**Authors:** Nan Wang, Tingting Liu

**Affiliations:** 1 School of Food Science and Engineering, Jilin Agricultural University, Changchun 130118, China; 18943920137@163.com; 2 Key Laboratory of Technological Innovations for Grain Deep-Processing and High-Effeciency Utilization of By-Products of Jilin Province, Changchun 130118, China; 3 Engineering Research Center of Grain Deep-Processing and High-Effeciency Utilization of Jilin Province, Changchun 130118, China

**Keywords:** curcumin, polysaccharide-based hydrogel, nanoparticles-in-hydrogel drug delivery system, pH-/temperature-responsive, colon-targeted release, ulcerative colitis

## Abstract

In ulcerative colitis (UC), the therapeutic efficacy of nanoparticle (NP)-based drug delivery systems is limited by premature drug release, uptake or degradation of NPs during their passage through the harsh gastrointestinal tract (GIT) environment, poor colon targeting, and rapid NP clearance caused by diarrhea symptoms. This study focused on designing an advanced spatiotemporally controlled nanohybrid hydrogel drug delivery system to overcome these challenges. We developed a pH- and temperature-responsive polysaccharide-based hydrogel composed of chitosan (CS), β-glycerol phosphate disodium salt pentahydrate (GP), hydroxypropyl cellulose (HPC), and collagen type I (Col I), designated as CS/HHPC/Col I-GP. The hydrogel exhibited a dense and uniform porous reticular structure, with an average pore diameter of 127.45 ± 2.22 μm. The equilibrium swelling ratio of the CS/HHPC/Col I-GP was determined to be 32.10 ± 1.11 g/g, indicating excellent swelling capacity and sustained structural stability over 6 h—making it suitable for sustained drug release in the intestinal tract. Then, the prepared curcumin nanoparticles (CurNPs) were encapsulated into the CS/HHPC/Col I-GP hydrogel to form the CS/HHPC/Col I-GP-CurNPs composite. The polysaccharide-based hydrogel shell of the formulation withstood harsh gastrointestinal conditions, enabled targeted adhesion to the colon, and was specifically degraded by colonic enzymes. The CurNPs released in the colon benefit from their negatively charged characteristics, enabling accumulation at the positively charged inflamed sites and achieving sustained Cur release. The results of the gastrointestinal digestion simulation experiment showed that the cumulative release of CS/HHPC/Col I-GP-CurNPs was only 12.33 ± 2.17% in simulated gastric fluid (SGF) and reached 96.91 ± 1.98% in simulated colonic fluid (SCF) after 60 h. Cell and animal experimental data confirmed that the formulation significantly alleviated colitis symptoms by modulating the repolarization of pro-inflammatory M1 macrophages to anti-inflammatory M2 phenotypes and deactivating the TLR4/MyD88/NF-κB pathway. Furthermore, the integrity of the intestinal mucosal barrier and the gut microbiota were enhanced. This study provides a promising strategy for the oral drug treatment of UC.

## 1. Introduction

UC is a chronic, idiopathic inflammatory disease characterized by recurrence and higher cancer risk, posing a significant burden on global healthcare systems [1,2,3]. Existing treatment strategies primarily rely on aminosalicylates, glucocorticoids, immunosuppressants, and biologics [4,5]. Nevertheless, these therapies are often associated with severe side effects [6,7]. Moreover, such interventions generally depend on intravenous and rectal administration methods, both of which have inherent drawbacks [8]. Intravenous delivery requires administration by a trained professional, whereas rectal administration may lead to mucosal irritation and considerable discomfort for patients [9,10]. Thus, developing an oral delivery system for UC treatment that is effective, safe, and ensures high patient compliance is urgently needed.

Cur, a natural bioactive polyphenol from turmeric, is widely considered safe by the Food and Drug Administration (FDA) [11,12]. Research confirms that Cur has significant efficacy in the treatment of UC [13,14]. Cur has demonstrated an ability to ameliorate UC symptoms by inhibiting inflammatory signaling pathways, maintaining intestinal microbiota balance, repairing the intestinal epithelial barrier, and exerting antioxidant effects, among others [15,16]. However, the oral administration of Cur is subject to many limitations, including poor aqueous solubility, rapid metabolism, low stability in the gastrointestinal environment, and poor colon targeting, resulting in low utility incommensurate with its potential [17]. In order to overcome these limitations, researchers have been developing NP delivery systems to enhance the bioavailability of Cur. It is reported that drug-loaded NPs can passively target colitis tissue via enhanced epithelial permeability and retention effects [18]. Although these effects increase the accumulation of drug-loaded NPs in colitis tissue, their therapeutic efficacy remains limited by rapid NP clearance due to diarrhea, extensive degradation in the harsh GIT, uptake by Peyer’s patches in the small intestine, and poor colon targeting [7,19]. Therefore, advanced pharmacotherapeutic technologies are needed that can deliver NPs and their payloads efficiently and precisely to the colon following oral administration, enabling accumulation in inflamed colonic tissue for localized drug delivery and achieving safe, sustained therapeutic outcomes.

Hydrogels are biomaterials with clinically proven biocompatibility and biofunctionality; they have emerged as a promising solution for UC treatment by enabling spatial and temporal control over the pharmaceutical activity of existing anti-inflammatory drugs [20,21]. Specifically, hydrogels are water-swollen networks of highly cross-linked polymer units that can provide ample space for efficient drug loading [22]. Ideally, hydrogel-based delivery systems would stably adhere to ulcerated lesions to provide a physical protective barrier and then release encapsulated drugs for effective treatment [3,23]. Nevertheless, traditional hydrogels are unable to precisely deliver drugs to inflamed sites due to their poor targeting ability and are unsuitable for oral drug delivery in the treatment of colon diseases. Current research is increasingly focused on smart hydrogels that provide controlled drug release by changing their structure in response to environmental stimuli [24,25]. Natural polysaccharides are biocompatible, non-toxic, cost-effective, and are used in FDA-approved clinical applications [17]. Their unique features (e.g., pH responsiveness, stomach resistance, degradation by colon microbiota, and mucosal adhesion) impart hydrogel systems with crucial properties to overcome GIT barriers associated with oral administration and achieve colon-specific drug release [26,27]. As a result, the development of polysaccharide-based smart-responsive hydrogel drug delivery systems and their application in UC treatment has become a fascinating research topic. CS is a polycationic polysaccharide that is biocompatible, pH-sensitive, strongly adhesive, and susceptible to degradation by specific enzymes secreted by colonic microbiota [3]. CS and its degradation products have been shown to inhibit the NF-κB pathway and inducible nitric oxide (NO) synthase; this in turn decreases NO production, reduces oxidative stress, and exerts anti-inflammatory effects [28,29]. Due to its advantages, CS is regarded as a promising UC-targeted carrier material for oral drug administration [30]. Notably, CS readily undergoes gelation and has been combined with GP to develop thermosensitive hydrogels (CS-GP) [31]. The CS-GP solution stays in liquid form at room temperature, allowing for easy oral administration. As the temperature increases to 37 °C, the solution transforms into a semi-solid gel. The CS component in CS-GP interacts with intestinal mucin via electrostatic forces, ensuring targeted drug release and effective therapy at inflamed colon sites [32]. However, these forces are unstable, and the ionic bonds between oppositely charged groups can be easily disrupted by intestinal peristalsis, reducing adhesive strength [10]. Additionally, hydrogels of this type have been reported to have low stability and poor mechanical properties, which can lead to low entrapment efficiency and abrupt drug release [33]. These disadvantages can be overcome by blending the hydrogel with a polymer that has better mechanical properties than CS and can produce a drug delivery system with better adhesive properties. HPC is a derivative of cellulose with nontoxicity, low equilibrium moisture content, and excellent bioadhesion [34]. HPC has been widely used in pharmaceutical formulations, such as an adhesive for oral tablets, hydrogel bases, and drug-loaded capsule materials [35]. Moreover, HPC is also a prebiotic dietary fiber, and its positive effects on the GIT have been explored [36]. Li et al. found that HPC alleviated colitis induced by dextran sulfate sodium (DSS) in mice by modulating gut microbiota [37]. Indeed, introducing HPC into CS-GP hydrogels allows the components to form an integrated polymer network through intermolecular forces, thereby improving the bioadhesive, mechanical, and functional properties of the original hydrogels [35]. Recent clinical evidence has suggested that colonic mucosal healing could serve as a reliable indicator of UC treatment efficacy [38]. Col, a natural polymer and key structural component of the extracellular dermal matrix, has been demonstrated to be a good matrix for mucosal ulcer repair [39,40]. Col I is the predominant type of Col in humans, and is a popular choice for hydrogels due to its ability to self-polymerize into gel networks in vitro. Additionally, Col I contains abundant functional groups, including –COOH, –OH, and –NH_2_, which can enhance hydrogel adhesion through hydrogen bonding interactions with polysaccharide compounds [41]. Considering these advantages, Col I-based hydrogels can serve as drug carriers and reconstructive matrices for damaged intestinal mucosa [42].

Given these considerations, a pH- and temperature-responsive polysaccharide-based hydrogel composed of CS, GP, HPC, and Col I was developed. The Cur-loaded NPs, which were previously prepared by our team using high hydrostatic pressure-treated zein and pectin, were encapsulated into the hydrogel [43]. This advanced spatiotemporally controlled nanohybrid hydrogel integrates the advantages of NPs and hydrogels, offering a synergistic strategy for drug delivery in UC and aiming to overcome the limitations associated with using either NPs or hydrogels alone as drug delivery systems in current research [17]. For example, Chen et al. prepared astaxanthin nanoparticles via a self-assembly method using whey protein isolate-dextran conjugates modified with (3-carboxypentyl) (triphenyl) phosphonium bromide [44]; Subsequently, lipoic acid modified hyaluronic acid was coated on the surface of the nanoparticles by double emulsion evaporation method for UC treatment. Li et al. fabricated oral administration of inflammatory microenvironment-responsive carrier-free infliximab nanocomplex with a high drug loading (>90%), colitis-targeting, and bio-response controlled release for UC treatment [45]. Wang et al. reported a facile prepared self-assembled supramolecular nanoparticle with pH-dependent reversible assembly based on natural polyphenol tannic acid and poly (ethylene glycol) containing polymer for oral anti-TNF-α antibody delivery in the treatment of inflammatory bowel disease [46]. However, when utilized alone as drug delivery vehicles, NPs have various drawbacks—such as their absorption in the upper gastrointestinal tract and premature drugs released—which can compromise their colon-targeting capability [47]; NPs are distributed indiscriminately, have a brief residence time, are taken up by systemic circulation for early evacuation from the body, and are inactivated [48]. Li et al. fabricated a thin shell hydrogel microcapsule with bis-crosslinking network of Ca^2+^ ion-mediated and UV-induced C-C covalent by microfluidic technique for the oral delivery of antibodies [49]. Moreover, Ding et al. developed an oral colon-targeted konjac glucomannan hydrogel construed by hostguest mediated noncovalent cross-linking of cucurbit uril for UC treatment [50]. However, due to the hydrophilic network structure of hydrogels, they are not suitable—when used alone—as drug delivery vehicles for hydrophobic components [51]. Moreover, cumbersome preparation steps, the sources of original materials are complex and expensive and unfavorable biosafety will quietly restrict their application and translation in clinical [23]. To date, few studies have investigated the development of distinct drug delivery system combinations for oral administration in UC. Therefore, the development of a spatiotemporally controlled nanohybrid polysaccharide-based hydrogel drug delivery system was highly innovative and offered the following advantages: (i) pH- and temperature responsiveness, enabling the gel solution to transition into a semi-solid hydrogel after entering the human body and effectively protecting Cur-loaded NPs from the harsh GIT environment; (ii) enhanced mechanical properties with improved adhesion and retention in the colon, enabling the hydrogel to resist gastrointestinal expulsion and strongly adhere to the colonic mucosa, thus avoiding rapid excretion of Cur-loaded NPs caused by diarrhea; (iii) colonic enzyme responsiveness and colon inflammation targeting, which enabled the drug to be efficiently and accurately delivered, with the hydrogel gradually degraded by microbial secretory enzymes within the colon, exposing the internal Cur-loaded NPs; the negatively charged Cur-loaded NPs subsequently aggregated around colonic epithelial cells that highly express the positively charged transferrin, especially when the mucus layer was compromised in UC, thus providing high local drug concentrations to inflamed intestinal areas for maximized efficacy; and (iv) the delivery carrier matrix and Cur worked synergistically to treat UC by modulating inflammatory factors, repairing damaged colonic mucosa, modulating redox balance and intestinal flora, and enhancing short-chain fatty acid (SCFA) production. Subsequently, the gelation time, porosity, mechanical strength, FTIR spectra, SEM morphology, rheological properties, swelling behavior, viscosity, and encapsulation efficiency of the nanohybrid dual-responsive polysaccharide-based hydrogel drug delivery system were characterized, and its drug release profile was evaluated under different pH conditions and temperatures simulating those of the GIT. After physicochemical characterization, the therapeutic efficacy of the formulation was evaluated in an inflammatory cell model using lipopolysaccharide (LPS)-stimulated RAW264.7 macrophages and in a DSS-induced UC mouse model. Overall, the novel spatiotemporally controlled nanohybrid polysaccharide-based hydrogel prepared in this work demonstrates great potential as a safe and efficient colon-targeted drug delivery system for UC treatment.

## 2. Results and Discussion

### 2.1. Gelation Time, Porosity, and Mechanical Strength of the Dual-Responsive Polysaccharide-Based Hydrogels

**Figure 1:**
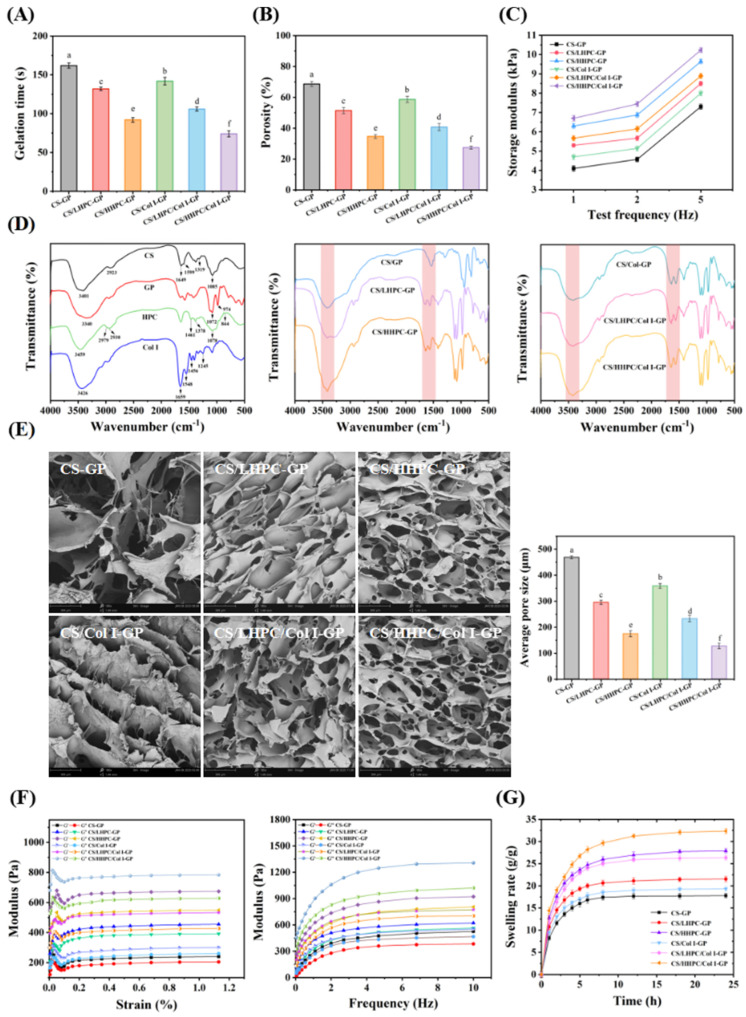
Characterization of different dual–responsive polysaccharide–based hydrogels. (**A**) Gelation time, (**B**) porosity, (**C**) mechanical strength, (**D**) FTIR spectra, (**E**) SEM images and average pore diameter, (**F**) rheological analysis, and (**G**) swelling rate. Significant differences in values (*p* < 0.05) are denoted by distinct letters (a–f).

The hydrogel gelation time was measured using the inverted vial method (Figure 1A). At 4 °C, the six gel solutions were able to flow. At 37 °C, the solutions underwent gelation within different time intervals, with the following order: CS/HHPC/Col I-GP (74.34 ± 4.55 s) < CS/HHPC-GP (92.02 ± 1.40 s) < CS/LHPC/Col I-GP (106.17 ± 2.79 s) < CS/LHPC-GP (132.50 ± 2.93 s) < CS/Col I-GP (142.11 ± 5.18 s) < CS-GP (162.69 ± 3.33 s). It was evident that the addition of HPC and Col I helped to shorten the gelation time. Because CS contains numerous amino and hydroxyl groups, it can form hydrogen bonds with the hydroxyl groups in HPC during intermolecular interactions. This increases the crosslinking density between polymer molecules, forming a more uniform and dense gel network structure, thereby accelerating the gelation process [35]. Col I also contains numerous polar groups, such as hydroxyl, amino, and carboxyl groups. These groups can interact with CS through hydrogen bonds and electrostatic interactions, which is conducive to increasing the crosslinking density of the gel system, ultimately shortening gelation time [52]. Additionally, the gelation time showed a negative correlation with the HPC concentration; thus, CS/HHPC/Col I-GP exhibited the shortest gelation time.

Figure 1B shows that CS-GP had the highest porosity, while CS/HHPC/Col I-GP had the lowest. In this study, the incorporation of HPC and Col I reduced the porosity of the hydrogel, with higher HPC concentrations further reducing the porosity. This could be due to the formation of more substantial and rigid hydrogel structures. This enhanced strength and rigidity may hinder pore formation or expansion, resulting in smaller pore sizes and reduced porosity [53]. A similar trend was noted by other authors in different hydrogel systems [48,54], including the phenomenon of faster gelation times and lower porosities at higher component concentrations, which is mainly attributed to the enhanced crosslinking density between polymer molecules.

To better protect the materials or drugs encapsulated within a hydrogel, a hydrogel carrier matrix must be sufficiently mechanically stable to withstand in vivo stresses [55,56]. As shown in Figure 1C, the incorporation of HPC and Col I markedly enhanced the mechanical strength of the hydrogel, with higher HPC concentrations yielding greater strength. This may result from functional groups such as hydroxyl groups, amino groups, and carboxyl groups in HPC and Col I, which generate intermolecular interaction forces with the CS-based hydrogel, leading to a higher degree of internal crosslinking, thus enhancing gel network stability and consequently improving mechanical properties [52,57]. Moreover, an increase in the testing frequency led to a corresponding enhancement in the hydrogel’s strength. The CS/HHPC/Col I-GP containing a high HPC concentration exhibited the greatest intensity at 5 Hz. The mechanical reinforcement of CS-based hydrogels by incorporating HPC has been previously reported [35].

### 2.2. FTIR Spectra and Morphology of the Dual-Responsive Polysaccharide-Based Hydrogels

The chemical structures of CS, GP, HPC, Col I, and the prepared hydrogels were comprehensively analyzed using FTIR. The results are shown in Figure 1D. The broad peak at 3401 cm^−1^ in the CS spectrum corresponded to O–H and N–H stretching vibrations, which were attributed to the polysaccharide structure [27]. The peak at 2923 cm^−1^ corresponded to the C–H symmetric stretching vibration. The absorption peaks at 1649 cm^−1^ and 1599 cm^−1^ were characteristic of amide I (C=O stretching) and amide II (N–H bending), respectively, confirming the presence of amino groups in CS [32]. The band at 1319 cm^−1^ corresponded to the C–O–H bending vibration, and the C–O stretching vibration appeared at 1085 cm^−1^. In the GP spectrum, the band at 3340 cm^−1^ was attributed to the O–H stretching vibration, which appeared broad owing to intermolecular hydrogen bonding involving the hydroxyl group. The asymmetric stretching vibration peak of –PO_4_^3−^ was observed at 1072 cm^−1^, and its symmetric stretching vibration peak appeared at 974 cm^−1^ [25]. The HPC spectrum showed a strong stretching vibration at 3459 cm^−1^, which corresponded to the characteristic hydroxyl group of cellulose [35]. The peaks at 2979 cm^−1^ and 2930 cm^−1^ corresponded to asymmetric and symmetric C–H stretching vibrations, respectively. The peak at 1461 cm^−1^ was attributed to the bending vibration of C–H due to the characteristic crystallization of cellulose, and the peak at 1378 cm^−1^ corresponded to the C–O–H bending vibration of the polymeric chain. Additionally, the bands observed at 1078 cm^−1^ and 844 cm^−1^ corresponded to alkoxy C–O and C–O–C stretching vibrations. Characteristic absorption bands of Col I were observed at 3426 cm^−1^ (amide A, N–H stretching coupled with hydrogen bonding), 1659 cm^−1^ (amide I, C=O stretching), 1548 cm^−1^ (amide II, N–H bending and C–N stretching), 1456 cm^−1^ (CH_2_ bending), and 1245 cm^−1^ (amide III, C–N stretching, N–H bending, and CH_2_ wagging from glycine backbone and proline side chains) [58]. Analysis of the CS-GP spectrum showed that the stretching vibrational peaks of the overlapped O–H and N–H at 3401 cm^−1^ shifted to a higher wavenumber and exhibited broadening, indicating hydrogen bond formation between the N–H groups in CS and the O–H groups in GP [31]. Additionally, due to hydrogen bonding between the O–H groups in CS and the –PO_4_^3−^ groups in GP, the C=O stretching vibration peak at 1649 cm^−1^ shifted to a lower wavenumber. The results indicated that a hydrogel network was formed between CS and GP through intramolecular and intermolecular hydrogen bonding. In the CS/LHPC-GP spectrum, the O–H band of pure CS and HPC shifted from 3401 cm^−1^ and 3459 cm^−1^, respectively, to 3417 cm^−1^ and broadened, indicating strong intermolecular hydrogen bonding between the functional groups (–NH_2_ and O–H) of the CS and HPC polymeric units [57]. The C=O stretching frequency (amide I band) shifted to a higher wavenumber compared to that in CS-GP, with the peak at 1076 cm^−1^ corresponding to C–O stretching vibrations. Furthermore, as the concentration of HPC increased, the O–H stretching vibration in the CS/HHPC-GP spectrum was enhanced and its range broadened, indicating strengthened hydrogen bond interactions between CS and HPC, thereby increasing the crosslinking density and forming a more uniform and dense gel network structure. The spectra of CS/Col I-GP, CS/LHPC/Col I-GP, and CS/HHPC/Col I-GP all displayed the main characteristic peaks of Col I, including the amide A, I, II, and III bands. The amide A band shifted from 3426 cm^−1^ to a higher wavenumber, and the amide II band also showed a blue shift. These shifts suggested the formation of intermolecular hydrogen bonds between the O–H groups in CS and HPC and the N–H groups in Col I [35]. Additionally, the increased intensities of the amide A and amide II bands with higher HPC concentration implied enhanced crosslinking between HPC and Col I [41]. The amide I band showed a red shift, suggesting an increase in C=O groups participating in hydrogen bonding, likely with the N–H groups of the same or adjacent chains. Overall, the strong interactions among CS, GP, HPC, and Col I caused shifts, changes in intensity, or disappearance of characteristic peaks, all of which are related to conformational changes between molecules and the formation of new bonds. These results indicated that CS-based hydrogels underwent crosslinking reactions with HPC through hydrogen bonding. Furthermore, CS formed hydrogen bonds with Col I, and formed weak electrostatic interactions between the side chain groups with opposite charges. These intermolecular interactions collectively contributed to the formation of a stable and dense gel network structure in CS/HHPC/Col I-GP, which facilitated the loading and delivery of drugs.

Figure 1E shows that the hydrogels clearly exhibited a three-dimensional porous structure with interconnected pores, which contributed to the loading and release of CurNPs; provided channels for the exchange of oxygen, carbon dioxide, and nutrients; and created an ideal microenvironment for cell growth and tissue remodeling [59]. A comparison of the microstructure among the six hydrogels showed that CS-GP exhibited a looser structure with large, irregularly distributed pores and an average pore diameter of 468.66 ± 2.45 μm. The addition of HPC and Col I led to a denser and more uniform pore structure within the hydrogels, and the average pore diameter decreased significantly. This phenomenon was attributed to the intermolecular interactions among HPC, Col I, and CS-based hydrogels, which enhanced the hydrogels’ crosslinking density [35]. With increasing HPC concentration, the hydrogel pore size decreased, suggesting stronger interactions among the hydrogel components. Therefore, the CS/HHPC/Col I-GP exhibited the densest and most uniform porous reticular structure, with an average pore diameter of 127.45 ± 2.22 μm. These findings indicated that the pore density of the hydrogel’s gel network was controlled by the extent of crosslinking [41].

### 2.3. Rheological Properties of the Dual-Responsive Polysaccharide-Based Hydrogels

The rheological properties of the hydrogels were analyzed by strain and frequency sweep measurements (Figure 1F). The storage modulus (G′) value reveals the elastic properties of the hydrogels, and the loss modulus (G″) value reveals the viscous properties of the hydrogels [30]. Throughout the tests, the G′ and G″ values of all groups remained uncrossed, with G′ dominant, exhibiting classical hydrogel behavior [60]. An oscillatory strain sweep was conducted on the hydrogels at 1 Hz with a strain range from 0.01% to 1.2%. Under low strain, both G′ and G″ exhibited instability, initially increasing and then decreasing. As the strain level increased, G′ and G″ gradually stabilized, indicating excellent mechanical integrity [61]. Furthermore, in the strain range, the G′ value consistently exceeded the G″ value, confirming that the hydrogels exhibited typical elastic (solid-like) properties, with high mechanical strength and excellent shape retention [24]. The frequency sweep data indicated that, at a fixed strain of 1% and frequencies varying from 0.01 Hz to 10 Hz, both G′ and G″ exhibited frequency-dependent behavior, indicating a stable hydrogel crosslinked network structure [62]. The G′ value of the hydrogels exceeded the G″ value, further confirming their elastic behavior within the tested frequency range. As the relative amounts of HPC and Col I gradually increased, their G′ and G″ values also became larger, with the G′ value of CS/HHPC/Col I-GP even reaching 1300 Pa. The main reason for this phenomenon was that increasing the polymer concentration in the hydrogel led to more extensive crosslinking, forming a denser network with higher mechanical strength [63]. Overall, the CS/HHPC/Col I-GP exhibited good elasticity, a stable structure, and some resistance to deformation and flow, which contributed to targeted drug release in the inflamed intestine.

### 2.4. Swelling Behavior of the Dual-Responsive Polysaccharide-Based Hydrogels

Swelling behavior of the hydrogels significantly influences controlled drug release in the body. Figure 1G shows the hydrogel swelling behavior in PBS (pH 7.4) at 37 °C. All the hydrogels exhibited a degree of swelling and remained intact. The swelling ratio of the hydrogels increased rapidly over time and reached swelling equilibrium within 15–20 h, after which the swelling rate remained stable, indicating that the hydrogels had excellent dimensional stability [56]. The rapid diffusion of water molecules into the hydrogels was driven by the high water uptake capacity of the hydrophilic groups in CS, HPC, and Col I, as well as by the osmotic pressure difference between the interior and exterior of the hydrogels. Water molecules were then trapped within the spaces between the polymer chains through hydrogen bonding, causing the hydrogels to swell with water [64]. As time increased, the osmotic pressure difference between the interior and exterior gradually decreased and tended to reach a balanced state. Macroscopically, this was manifested as a slower swelling rate of the hydrogels that eventually stabilized [65]. Research has shown that the chemical composition, structure, and crosslinking density determine the degree of hydrogel swelling [66]. The equilibrium swelling rate of CS-GP, CS/LHPC-GP, CS/HHPC-GP, CS/Col I-GP, CS/LHPC/Col I-GP, and CS/HHPC/Col I-GP was determined to be 17.72 ± 0.62 g/g, 21.45 ± 0.95 g/g, 27.74 ± 0.83 g/g, 19.25 ± 1.09 g/g, 26.25 ± 0.67 g/g, and 32.10 ± 1.11 g/g, respectively, indicating that all the hydrogels exhibited good water absorption. The swelling rate increased with increasing relative amounts of HPC and Col I, consistent with previous research findings [35]. On the one hand, this increased swelling rate was due to the hydrogels’ large specific surface area and abundant interconnected pores, which allowed water to quickly penetrate into the interior of the hydrogel [65]. On the other hand, HPC and Col I contained –COOH and –OH groups, which acted as acidic pendant groups between the chains that helped trap water inside their structure; additionally, with an increasing degree of crosslinking in the hydrogels, the internal structure became denser, which inhibited water evaporation [67]. These findings strongly supported the conclusion that CS/HHPC/Col I-GP had excellent swelling performance and maintained stability for up to 6 h, rendering it suitable for sustained drug release in the intestinal tract.

### 2.5. Biodegradation Behavior of the Dual-Responsive Polysaccharide-Based Hydrogels

The biodegradation behavior of the hydrogels was evaluated under simulated GIT physiological conditions. As shown in Supplementary Figure S1A, the weight loss of all the hydrogels increased with increasing incubation time, indicating biodegradation. CS-GP showed rapid degradation in SGF, with a degradation rate of 76.18 ± 3.45% after 24 h. This was mainly attributed to CS containing abundant –NH_2_ and –OH groups, which easily undergo protonation in acidic environments, making the hydrogels susceptible to degradation at the pH of SGF [68]. However, incorporating HPC and Col I effectively inhibited the degradation of the hydrogels in SGF; in particular, CS/HHPC/Col I-GP was relatively stable in SGF, degrading by only 32.67 ± 3.16% after 24 h. This phenomenon could be explained by enhanced crosslinking reactions among Col I, HPC, and CS-based hydrogels through hydrogen bonding and electrostatic interactions, forming a compact gel network structure, thus protecting the integrity of the hydrogels in gastric acid to some extent [35]. Notably, the degradation rates of all the hydrogels in SCF were relatively high, indicating their high sensitivity to alkaline environments [17]; among them, CS/HHPC/Col I-GP degraded by 92.69 ± 2.59% after 24 h. This phenomenon can also be explained by the fact that, after exposure of the hydrogels to the simulated colonic environment, CS was specifically degraded by colonic enzymes, leading to the collapse and degradation of the hydrogel structure. A previous study reported that a hydrogel formed from a furfural-functionalized CS-mannose polymer also showed a similar biodegradation profile [3]. These results suggested that CS/HHPC/Col I-GP could remain stable in the stomach after oral administration but undergo continuous and enhanced degradation in the colon, contributing to targeted drug delivery to the colon.

### 2.6. Adhesion Effect of the Dual-Responsive Polysaccharide-Based Hydrogels

One of the important factors for the hydrogel delivery system to play a role in UC treatment is its ability to adhere to the inflamed site in the colon and continuously release drugs. Supplementary Figure S1B shows the hydrogel viscosity as indicated by the ball’s rolling distance on the hydrogel surface. The results indicated significant differences in the viscosities of hydrogels with varying HPC concentrations (*p* < 0.05). HPC increased the physical adhesion of the hydrogels, and as the HPC concentration increased, the hydrogel viscosity correspondingly increased. To assess hydrogel adhesion to colon tissue, we measured the volume of hydrogel that spread over the surface of colon tissue (Supplementary Figure S1C). All the hydrogels showed some degree of adhesion to colon tissue, with higher HPC concentrations enhancing the adhesive properties of the hydrogel. This is because HPC forms hydrogen bonds with molecules on the colonic mucosa surface, thereby improving adhesion [25]. Supplementary Figure S1D shows the hydrogel adhesion force measured after complete gelation on colon tissue. The gel solutions transformed into a semi-solid form and adhered to the colon tissue at 37 °C, thereby avoiding rapid drug clearance following administration due to diarrhea symptoms caused by UC. The findings indicated that HPC could enhance the adhesion between the hydrogel and colon tissue, with greater adhesion strength observed at higher HPC concentrations. Additionally, the presence of Col I also enhanced the viscosity and adhesion performance of the hydrogels. This is because both HPC and Col I are viscous and have been used as thickeners in various applications, such as in medicine, food, and cosmetics [69,70]. A previous study demonstrated that HPC and Col I substantially increased the tissue adhesion of CS-based thermosensitive hydrogels, enabling them to gel during endoscopic mucosal dissection and ensuring sustained adherence to the wound site post-surgery, thereby minimizing both intraoperative and postoperative complications [35]. As a result, CS/HHPC/Col I-GP showed the strongest adhesion.

### 2.7. Encapsulation Efficiency and Zeta Potential of the Dual-Responsive Polysaccharide-Based Hydrogels Loaded with Cur Nanoparticles

**Figure 2:**
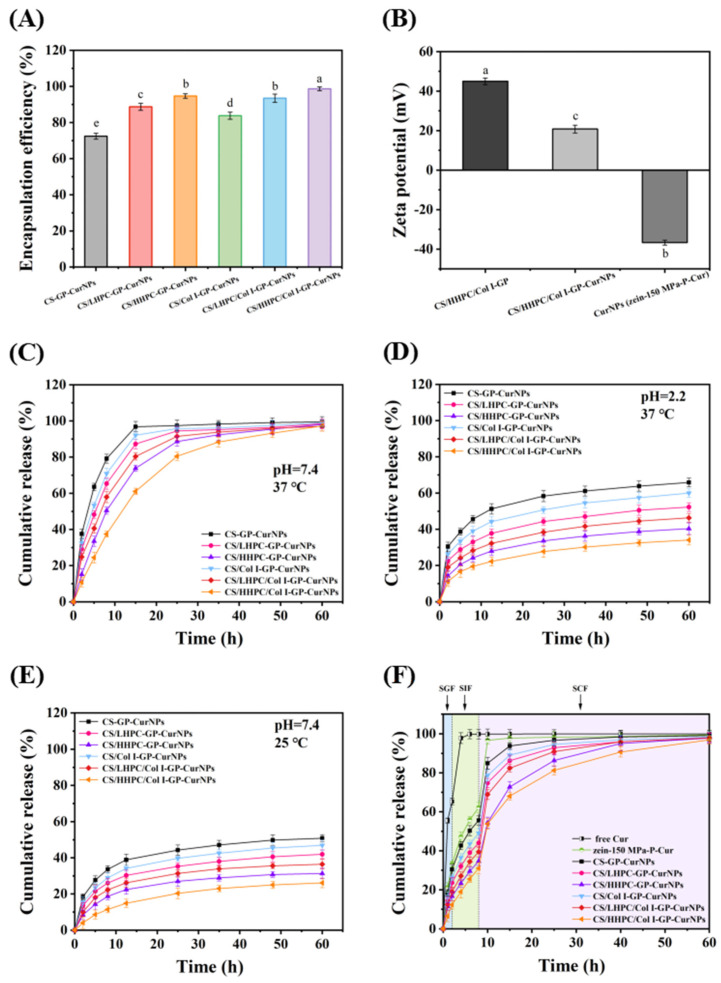
(**A**) Encapsulation efficiency, (**B**) zeta potential, and cumulative drug release of different dual–responsive polysaccharide–based hydrogels loaded with CurNPs under (**C**) pH 7.4 and 37 °C, (**D**) pH 2.2 and 37 °C, (**E**) pH 7.4 and 25 °C, and (**F**) in a simulated GIT environment. Significant differences in values (*p* < 0.05) are denoted by distinct letters (a–e).

In this study, Cur-loaded NPs (zein-150 MPa-P-Cur) were successfully prepared using antisolvent precipitation and incorporated into the hydrogels. High encapsulation efficiency is especially important for chemically unstable drugs. Figure 2A shows the Cur encapsulation efficiency in the hydrogels loaded with CurNPs, indicating that the chemical composition of the hydrogels affected Cur encapsulation efficiency. By incorporating HPC and Col I, the encapsulation efficiency of the hydrogels loaded with CurNPs was significantly increased (*p* < 0.05), and the highest encapsulation efficiency of 98.67 ± 1.86% was observed for CS/HHPC/Col I-GP-CurNPs. These findings indicated that the formulation successfully and efficiently encapsulated Cur. This was due to the hydrogel matrix’s abundant functional groups, especially –OH and –COOH, which could interact intermolecularly with the core–shell structure of the Cur-loaded NPs [71]. This phenomenon also appeared to be related to the high crosslinking density of the hydrogels, which effectively prevented the outward diffusion of Cur-loaded NPs and firmly confined them within the gel network during the formation of the nanohybrid hydrogel delivery system, thereby significantly increasing encapsulation capacity [72]. Furthermore, the zeta potential was assessed to further investigate the interaction between Cur-loaded NPs and the hydrogel. CS/HHPC/Col I-GP exhibited a strong positive charge due to CS, while Cur-loaded NPs were negatively charged. Thus, a change in potential occurred when Cur-loaded NPs were coated with CS/HHPC/Col I-GP. Figure 2B shows that CS/HHPC/Col I-GP exhibited a high positive zeta potential of 45.01 ± 1.34 mV, while CS/HHPC/Col I-GP-CurNPs showed a reduced value of 20.79 ± 2.55 mV, indicating that the Cur-loaded NPs were successfully encapsulated. Meanwhile, this also indicated the existence of electrostatic interactions between Cur-loaded NPs and the hydrogel, which arose from the negatively charged –COOH groups in the pectin shell of the Cur-loaded NPs and the positively charged –NH_2_ groups in the CS of the hydrogel matrix [73].

### 2.8. Drug Release from Dual-Responsive Polysaccharide-Based Hydrogels Loaded with Cur Nanoparticles at Different pH Values and Temperatures

To investigate the pH-responsive and temperature-responsive drug release behaviors of the hydrogels loaded with CurNPs, in vitro simulation experiments were conducted under the following conditions: pH 7.4 at 37 °C, pH 2.2 at 37 °C, and pH 7.4 at 25 °C. As shown in Figure 2C, the CS-GP-CurNPs demonstrated a rapid initial release, particularly at pH 7.4 and 37 °C, with the cumulative release reaching 37.54 ± 2.02% within 2 h and almost complete release observed by 15 h. In contrast, the other hydrogels loaded with CurNPs showed a slower initial release; CS/HHPC/Col I-GP-CurNPs released 10.88 ± 1.11% after 2 h, reaching 61.05 ± 2.38% at 15 h. This differential release behavior can be mechanistically attributed to a densely crosslinked gel network with a compact structure forming after HPC and Col I were incorporated into the CS-based hydrogels, which increased steric hindrance and reduced buffer solution penetration, thereby suppressing the initial Cur burst release and enabling a sustained release pattern [74]. The cumulative release of the hydrogels loaded with CurNPs ranked in order of magnitude were as follows: CS/GP-CurNPs > CS/Col I-GP-CurNPs > CS/LHPC-GP-CurNPs > CS/LHPC/Col I-GP-CurNPs > CS/HHPC-GP-CurNPs > CS/HHPC/Col I-GP-CurNPs. The release profiles of the hydrogels loaded with CurNPs at pH 2.2 and 37 °C are shown in Figure 2D, with a similar trend as that of the release profile at pH 7.4 and 37 °C; however, the released fraction of Cur from all samples at pH 2.2 and 37 °C was approximately half of that observed at pH 7.4 and 37 °C. This phenomenon demonstrated the pH-responsive behavior of the hydrogels loaded with CurNPs. The carboxylic acid groups of the hydrogels were easily deprotonated at higher pH levels, such as 7.4, generating ions that induced electrostatic repulsion, thereby increasing the swelling of the hydrogel structure and allowing the entrapped CurNPs to escape and be released into the surrounding medium, thus facilitating the release of Cur. At low acidic pH, minimal Cur was released because the hydrogel functional groups were protonated, causing the hydrogels to become partially swollen or collapsed, and the compact gel structure trapped the CurNPs within the polymeric network [48]. The release profiles of the hydrogels loaded with CurNPs at pH 7.4 and 25 °C are shown in Figure 2E, with a similar trend as that of the release profile at pH 7.4 and 37 °C; however, the released fraction of Cur from all samples at pH 7.4 and 25 °C was approximately one-third of that observed at pH 7.4 and 37 °C. This phenomenon was directly related to the phase transition behavior of the hydrogels under temperature changes [75], indicating the temperature-responsive behavior of the hydrogels loaded with CurNPs. At 25 °C, the thermal motion of molecules was relatively low, and the hydrogen bonding between HPC, CS, and water molecules dominated, enabling the gel solution system to maintain a flowable liquid state. Due to their low diffusion rate, CurNPs remained within the gel solution system, ultimately delaying the release of Cur. As the temperature rose, the thermal motion of the molecules intensified, the hydrogen bonds between water molecules and polymer molecules broke, and the hydrophobic interactions between polymer molecules strengthened, causing significant entanglement and aggregation of the molecular chains, which subsequently formed a strong gelation network structure. When the temperature rose to 37 °C (close to or exceeding the minimum critical dissolution temperature of the hydrogel), the “squeezing effect” caused by the complete transformation of the sol into a semi-solid gel led to the rapid release of the encapsulated CurNPs, ultimately significantly enhancing the release rate of Cur [76]. These results clarified that the hydrogels loaded with CurNPs exhibited pH-responsive and temperature-responsive drug release behaviors; among them, CS/HHPC/Col I-GP-CurNPs was speculated to have good colon microenvironment-responsive properties (37 °C, pH 7.4).

### 2.9. In Vitro Drug Release of the Dual-Responsive Polysaccharide-Based Hydrogels Loaded with Cur Nanoparticles

The drug release from the hydrogels loaded with CurNPs was evaluated in a simulated GIT environment, with release profiles shown in Figure 2F. Free Cur was rapidly released in SGF and was completely released within 6 h, with a cumulative release of 99.82 ± 0.34%. When Cur was encapsulated in NPs (CurNPs, zein-150 MPa-P-Cur), its release rate was relatively reduced in SGF; however, in SCF, nearly complete release of Cur was observed within 2 h, reaching 96.67 ± 0.50%. This may result from the core–shell structure of the CurNPs, which was susceptible to uncontrolled degradation in gastrointestinal digestive juices; as a result, the CurNPs became unstable and prone to burst release upon entering the colonic fluid—a phenomenon that is unconducive to effective UC treatment [77]. In contrast, when the CurNPs were encapsulated in the hydrogels, sustained and enhanced release of Cur in the colon was achieved—driven by electrostatic interactions between the CurNPs and the hydrogels, as well as by the swelling properties of the hydrogels [25]. Specifically, all the hydrogels loaded with CurNPs demonstrated a relatively low Cur release in SGF; however, they displayed a sustained release in SCF, with a stable release rate that followed a linear pattern, mitigating the toxicity associated with burst release and providing a long-lasting therapeutic effect. This phenomenon can be explained as follows: in an acidic medium, the amino groups of the hydrogels were protonated, resulting in unswollen or collapsed hydrogels; the compact gel structure allowed the hydrogels to trap the CurNPs within the polymeric network, which ultimately decreased the release rate of Cur [78]. The carboxylic acid groups of the hydrogels were easily deprotonated at alkaline pH (e.g., 7.4), with the resulting ions generating electrostatic repulsion, which increased hydrogel swelling and allowed the entrapped CurNPs to escape into the surrounding medium, thereby facilitating Cur release [48]. At the same time, colon-specific enzymes effectively degraded the CS-based hydrogel scaffold, further facilitating the Cur release [79]. Notably, the cumulative release of CS/HHPC/Col I-GP-CurNPs was 12.33 ± 2.17% in SGF and reached 96.91 ± 1.98% in SCF after 60 h; these results suggested that it had optimal properties for colon-targeted drug delivery suitable for oral UC treatment due to its dense and stable gel network structure and excellent swelling performance.

The Cur release kinetics of the hydrogels loaded with CurNPs in the simulated GIT environment were analyzed using different mathematical models (Table 1). The released fractions from the hydrogels loaded with CurNPs fitted the Korsmeyer–Peppas model better, exhibiting correlation coefficients (R^2^) between 0.9766 and 0.9976, along with release exponents (n) all less than 0.5. These findings indicated that the Cur release followed Fickian diffusion behavior, primarily occurring due to the swelling or erosion of the hydrogel polymer network and Cur diffusion within the gel matrix, leading to the controllable release behavior of Cur [80]. Specifically, swelling and erosion are the key changes that occur in hydrogels immersed in the release medium (colonic fluid), directly affecting the release behavior of Cur from the hydrogel-based carrier. The swelling of hydrogels is a process in which they absorb solvent, leading to an increase in volume. By increasing the porosity and specific surface area of hydrogels, Cur can be diffused and released; the higher the degree of swelling, the faster the initial release rate may be. The erosion of hydrogels is a process in which the polymer network undergoes chemical degradation in the release medium, leading to the gradual breakdown of its structure; as the polymer network degrades, the Cur release pathway increases, which may lead to sustained release. In this study, the two acted synergistically; the hydrogel first rapidly released a portion of Cur through swelling and then continuously released Cur through erosion.

### 2.10. Cytotoxicity of CS/HHPC/Col I-GP-CurNPs

In view of these findings on the physicochemical properties of the dual-responsive polysaccharide-based hydrogels loaded with CurNPs, we selected CS/HHPC/Col I-GP-CurNPs for subsequent cell and animal experiments. Cytotoxicity assessment is essential for developing new drug delivery agents. Here, RAW264.7 macrophages were treated with LPS (0.25–8 μg/mL) or CS/HHPC/Col I-GP-CurNPs (1–100 μg/mL), and their cytotoxicity was evaluated using a CCK-8 assay. LPS at concentrations between 0.25 and 2 μg/mL exhibited no notable cytotoxic effects (*p* > 0.05) (Supplementary Figure S2A). When the LPS concentration increased to 4 μg/mL, cell viability significantly decreased (*p* < 0.05); at 8 μg/mL, the decrease was highly significant (*p* < 0.01). Notably, when cells were incubated with 2 μg/mL LPS, a decreasing trend in cell viability was observed, although no significant difference was found (*p* > 0.05). Overall, at 1 μg/mL, the cell viability was 96.16 ± 3.23%, and this concentration was selected for evaluating the treatment effects of CS/HHPC/Col I-GP-CurNPs against inflammation in subsequent studies. As shown in Supplementary Figure S2B, when cells were treated with CS/HHPC/Col I-GP-CurNPs (1–100 μg/mL) and LPS (1 μg/mL), cell viability initially decreased and then increased compared with the negative control, reaching a maximum of 101.64 ± 1.25% for CS/HHPC/Col I-GP-CurNPs at 10 μg/mL, suggesting that this concentration provided the optimal balance between efficacy and cellular compatibility. However, when the concentration of CS/HHPC/Col I-GP-CurNPs was above 10 μg/mL, cell viability significantly decreased (*p* < 0.01); thus, 10 μg/mL was chosen for subsequent cellular assays. These results suggested that CS/HHPC/Col I-GP-CurNPs had a certain restorative effect against LPS-induced macrophage damage.

### 2.11. Cellular Uptake of CS/HHPC/Col I-GP-CurNPs

**Figure 3:**
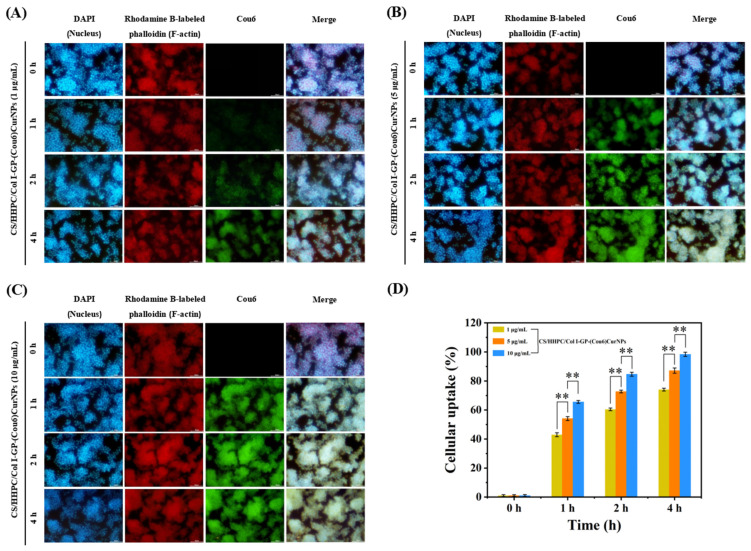
Cellular uptake of CS/HHPC/Col I-GP-CurNPs at different concentrations by RAW264.7 cells over various time periods. Fluorescence microscope images of the cellular internalization profiles of Cou6-labeled CS/HHPC/Col I-GP-CurNPs ((**A**): 1 μg/mL, (**B**): 5 μg/mL, (**C**): 10 μg/mL; scale bar: 50 μm). (**D**) Percentage of cells containing CS/HHPC/Col I-GP-(Cou6)CurNPs after incubation. Significance levels: ** *p* < 0.01.

Efficient uptake of delivery carriers by macrophages in inflamed lesions is essential for effective colitis treatment [13]. In this study, in vitro uptake studies of CS/HHPC/Col I-GP-CurNPs by activated RAW264.7 macrophages were performed. After CurNPs were labeled with the Cou6 dye (green fluorescence) to prepare CS/HHPC/Col I-GP-(Cou6)CurNPs and incubated with macrophages, nuclei were stained with DAPI (blue fluorescence), and cytoskeletons were labeled with rhodamine B-phalloidin (red fluorescence). Green fluorescence was observed in the cytoplasmic regions of RAW264.7 cells after 1 h of incubation with CS/HHPC/Col I-GP-(Cou6)CurNPs, probably due to the uptake of CS/HHPC/Col I-GP-(Cou6)CurNPs by RAW264.7 cells through endocytosis (Figure 3A–C). The green fluorescence intensity significantly increased with higher concentrations (1–10 μg/mL) and longer incubation times (1–4 h), indicating that the uptake of CS/HHPC/Col I-GP-(Cou6)CurNPs by RAW264.7 cells was dependent on both concentration and incubation time. The fluorescence results were further confirmed through quantitative analysis using FCM (Figure 3D). Notably, after 4 h of incubation, the cellular internalization efficiencies of different concentrations of CS/HHPC/Col I-GP-(Cou6)CurNPs were all above 70%. This was attributed to the electropositive nature of CS in CS/HHPC/Col I-GP-(Cou6)CurNPs, which interacted with the negatively charged cell membrane to enhance the adsorption and endocytosis of the hydrogel carrier matrix by RAW264.7 cells [81,82]. In summary, CS/HHPC/Col I-GP-CurNPs were easily taken up by RAW264.7 cells through a rapid and efficient intrinsic action, which made them suitable for drug delivery in UC treatment.

### 2.12. Wound Healing Capacity of CS/HHPC/Col I-GP-CurNPs

**Figure 4:**
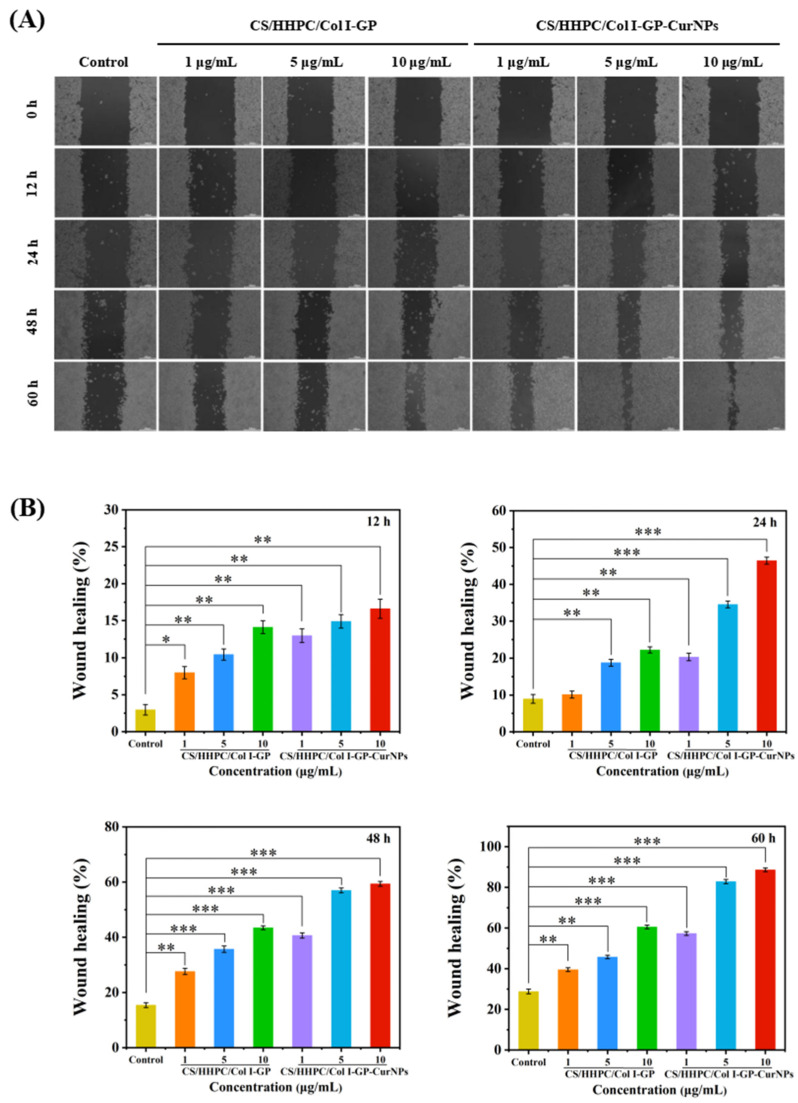
Cell scratch assay results. (**A**) Damaged cells were treated with different concentrations of CS/HHPC/Col I-GP and CS/HHPC/Col I-GP-CurNPs, and microscopic images of wound healing were captured at various time points (scale bar: 200 μm). (**B**) Quantitative analysis of wound healing after different treatments. Significance levels: * *p* < 0.05, ** *p* < 0.01, and *** *p* < 0.001.

The colonic epithelial barrier comprises multiple components, including colonic epithelial cells, colonic mucus, the mucosal immune system, and gut microbiota, which together provide efficient protection [83]. However, under the influence of certain external factors, such as physical damage or stimulation by chemical substances, the intercellular spaces may open, allowing harmful substances to penetrate into the colonic epithelial tissue, damage the epithelial cells, and thereby disrupt the barrier function of the colonic epithelium [84]. In this study, artificial scratches were used to simulate cell damage, and healing was monitored after treatment (Figure 4A,B). The CS/HHPC/Col I-GP and CS/HHPC/Col I-GP-CurNPs groups exhibited significantly narrower scratch widths in the cells compared to the control group. This phenomenon was attributed to Col I in the hydrogel matrix, whose RGD (Arg-Gly-Asp) sequence supports cell signaling, migration, and proliferation [39]. Additionally, the group treated with CS/HHPC/Col I-GP-CurNPs showed the smallest scratch area due to the sustained release of Cur, which reduced oxidative stress among cells and promoted better cell proliferation [85]. The cell scratch healing rates in the control group, the 10 μg/mL CS/HHPC/Col I-GP group, and the 10 μg/mL CS/HHPC/Col I-GP-CurNPs group were approximately 28.90 ± 1.15%, 60.53 ± 0.92%, and 88.64 ± 0.94% after 60 h, respectively, showing a significant difference. These findings showed the positive effects of CS/HHPC/Col I-GP-CurNPs on cell migration and wound healing processes, which may help repair a damaged colonic barrier in UC.

### 2.13. Cellular Antioxidant and Anti-Inflammatory Effects of CS/HHPC/Col I-GP-CurNPs

**Figure 5:**
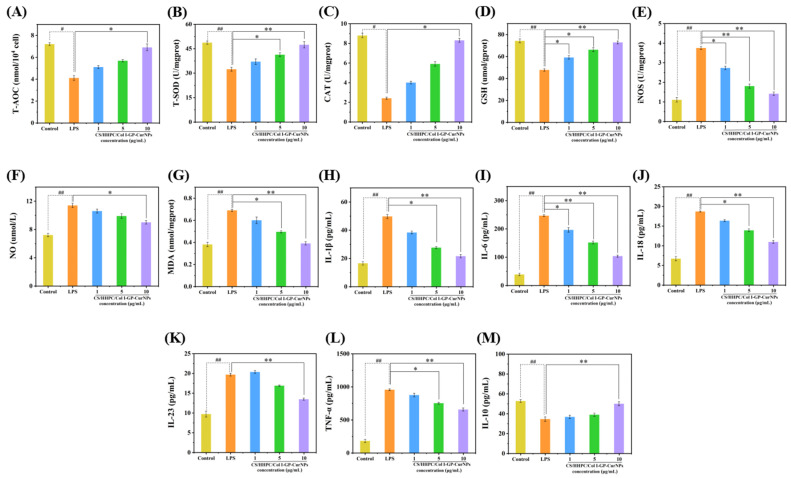
Cellular antioxidant and anti-inflammatory effects analysis. Levels of (**A**) T-AOC, (**B**) T-SOD, (**C**) CAT, (**D**) GSH, (**E**) iNOS, (**F**) NO, (**G**) MDA, (**H**) IL-1β, (**I**) IL-6, (**J**) IL-18, (**K**) IL-23, (**L**) TNF-α, and (**M**) IL-10 in RAW264.7 cells cultured with CS/HHPC/Col I-GP-CurNPs at different concentrations, measured by the corresponding assay kits. Significance levels: ^#^ *p* < 0.05 and ^##^ *p* < 0.01 vs. control group; * *p* < 0.05 and ** *p* < 0.01 vs. LPS group.

The antioxidant and anti-inflammatory effects of CS/HHPC/Col I-GP-CurNPs were assessed by measuring oxidative stress markers and inflammatory cytokines in LPS-induced RAW264.7 macrophages. The T-AOC reflects the ability to scavenge free radicals and inhibit lipid peroxidation. In macrophages stimulated with 1 μg/mL LPS, T-AOC levels significantly decreased compared to those in control macrophages (*p* < 0.05) (Figure 5A). However, in macrophages treated with CS/HHPC/Col I-GP-CurNPs for 24 h, T-AOC levels increased with increasing concentrations of CS/HHPC/Col I-GP-CurNPs; when the concentration of CS/HHPC/Col I-GP-CurNPs was 10 μg/mL, T-AOC levels increased by 68.29% relative to those in LPS-treated cells. Antioxidant enzymes, including T-SOD, CAT, and GSH, are essential for preserving redox balance and alleviating oxidative stress injury [86]. The levels of T-SOD, CAT, and GSH in the LPS group were markedly decreased compared with those in the control group (*p* < 0.05 and *p* < 0.01) (Figure 5B–D). However, after the macrophages were pre-incubated with CS/HHPC/Col I-GP-CurNPs for 24 h, the intracellular T-SOD, CAT, and GSH activities increased compared to those in LPS-treated macrophages; in particular, treatment with 10 μg/mL CS/HHPC/Col I-GP-CurNPs restored these levels to near those of the control macrophages. The iNOS activity was upregulated in LPS-injured cells, resulting in the production of the pro-oxidative marker NO and the end product of excessive lipid oxidation, MDA—both of which are key indicators of inflammation and cancer progression [87]. Compared with LPS treatment alone, the activity of iNOS decreased after pretreatment with CS/HHPC/Col I-GP-CurNPs, and the production of NO and MDA was significantly reduced (*p* < 0.05 and *p* < 0.01) (Figure 5E–G). Treatment with 10 μg/mL CS/HHPC/Col I-GP-CurNPs produced the most pronounced effect. The comprehensive analysis showed that CS/HHPC/Col I-GP-CurNPs had superior antioxidant properties, which alleviated oxidative stress in LPS-induced cellular inflammation.

Proinflammatory cytokines (IL-1β, IL-6, IL-18, IL-23, and TNF-α) and the anti-inflammatory cytokine IL-10 are well-known inflammatory markers used to monitor UC progression [88]. Therefore, the effects of CS/HHPC/Col I-GP-CurNPs on the levels of these factors were investigated in LPS-induced RAW264.7 macrophages. Figure 5H–L shows that LPS treatment remarkably increased the proinflammatory cytokine levels, indicating that LPS induced inflammation in the cells (*p* < 0.01). In contrast, after the macrophages were pre-incubated with CS/HHPC/Col I-GP-CurNPs, the secretion of these pro-inflammatory cytokines decreased, indicating that CS/HHPC/Col I-GP-CurNPs exerted an anti-inflammatory effect. The most significant reduction was observed in the 10 μg/mL CS/HHPC/Col I-GP-CurNPs treatment group (*p* < 0.01). Compared to LPS treatment alone, the levels of the anti-inflammatory cytokine IL-10 after pretreatment with CS/HHPC/Col I-GP-CurNPs exhibited a dose-dependent increase (Figure 5M). This was expected because CS/HHPC/Col I-GP-CurNPs slowly released Cur when co-incubated with macrophages, and Cur subsequently exerted excellent anti-inflammatory effects in cells via multiple pathways [89]. In summary, these findings demonstrated that CS/HHPC/Col I-GP-CurNPs effectively alleviated LPS-induced cell damage and exhibited significant in vitro antioxidant and anti-inflammatory effects.

### 2.14. Regulatory Effects on Macrophage Polarization of CS/HHPC/Col I-GP-CurNPs

**Figure 6:**
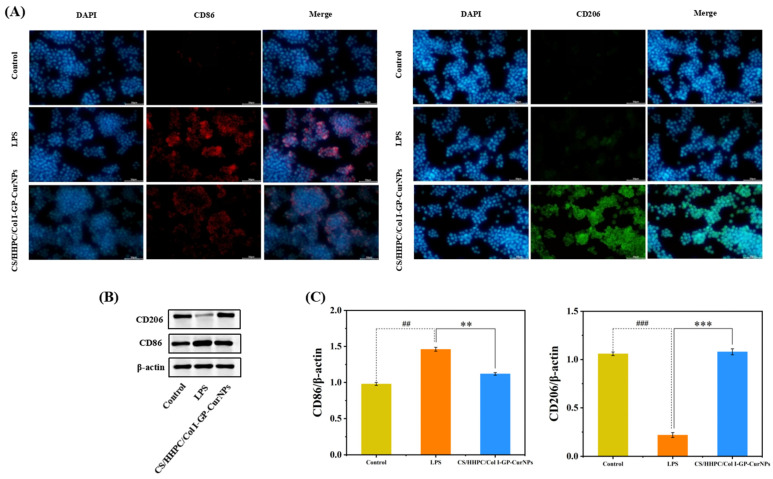
The influence of CS/HHPC/Col I-GP-CurNPs on macrophage phenotype switching. (**A**) Immunofluorescence staining images of surface biomarkers (scale bar: 50 μm). (**B**) Western blotting images of CD86 and CD206 in macrophages. (**C**) Protein expression levels of CD86 and CD206. Significance levels: ^##^ *p* < 0.01, and ^###^ *p* < 0.001 vs. control group; ** *p* < 0.01, and *** *p* < 0.001 vs. LPS group.

Restoring immune homeostasis by inducing macrophage polarization from pro-inflammatory M1 to anti-inflammatory M2 phenotypes is a promising therapeutic approach for UC [6]. The immunomodulatory effects of CS/HHPC/Col I-GP-CurNPs on macrophages stimulated by LPS were assessed through immunofluorescence staining. CD86 (red fluorescence) is widely recognized as a marker for M1 macrophages, and CD206 (green fluorescence) is frequently employed to identify M2 macrophages [3]. The results showed that CD86 and CD206 expression levels were not prominent in the control macrophages, indicating that most of these macrophages were in an inactive state (Figure 6A). Nevertheless, after LPS stimulation, CD86 expression (red fluorescence) became dominant, suggesting that macrophages were polarized toward the M1 phenotype [90]. Notably, after incubation with CS/HHPC/Col I-GP-CurNPs, LPS-induced macrophages exhibited reduced expression of CD86 and enhanced expression of CD206, signifying a shift toward M2 macrophages. The Western blotting results also showed a similar trend: in the CS/HHPC/Col I-GP-CurNPs group, CD86 protein expression was markedly reduced compared to the LPS-treated group (*p* < 0.01), while CD206 expression showed significant upregulation (*p* < 0.001) (Figure 6B,C). Within the UC microenvironment, macrophages are typically skewed toward the proinflammatory M1 phenotype and are accompanied by oxidative stress and inflammatory reactions. CS/HHPC/Col I-GP-CurNPs scavenged free radicals and downregulated M1 phenotype macrophages, leading to reduced secretion of pro-inflammatory cytokines and simultaneously increased anti-inflammatory cytokine secretion, facilitating M2 polarization and displaying robust immunomodulatory functions, which may further improve inflammatory disease symptoms [14].

### 2.15. Hemocompatibility of CS/HHPC/Col I-GP-CurNPs

Hemocompatibility is crucial for the in vivo application of CS/HHPC/Col I-GP-CurNPs. Supplementary Figure S3 shows that CS/HHPC/Col I-GP-CurNPs did not induce significant hemolysis after co-incubating with red blood cells. Hemolysis rates remained below 2.0% across all tested concentrations (0.125–2 mg/mL) after 4 h, well under the internationally recognized 5% standard [90]. This finding indicated that CS/HHPC/Col I-GP-CurNPs possessed good hemocompatibility, making them appropriate for further in vivo studies.

### 2.16. Biodistribution of CS/HHPC/Col I-GP-CurNPs in DSS-Induced UC Mice

**Figure 7:**
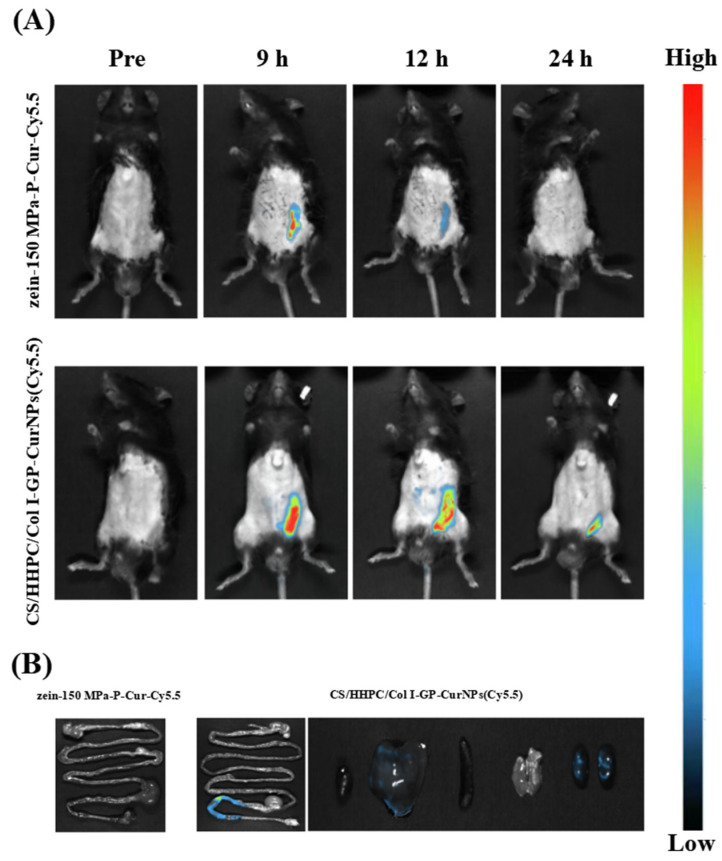
(**A**) In vivo fluorescence imaging of orally administered zein-150 MPa-P-Cur-Cy5.5 and CS/HHPC/Col I-GP-CurNPs(Cy5.5) in DSS-induced UC mice. (**B**) Representative fluorescence images of organs (intestines, heart, liver, spleen, lungs, and kidneys) after 24 h.

In light of these findings from the aforementioned in vitro experiments, we propose that CS/HHPC/Col I-GP-CurNPs can adapt to the complex motility, drastic pH changes in the GIT and adhere to the inflamed colon mucosa over a large area, ensuring sufficient intestinal retention for sustained Cur release. To validate this hypothesis, the biodistribution of CS/HHPC/Col I-GP-CurNPs(Cy5.5) in DSS-induced UC mice after oral administration was investigated using an IVIS imaging system. The results demonstrated that the fluorescence signals were detected at 9 h and 12 h post-administration in the DSS-induced UC mice treated with zein-150 MPa-P-Cur-Cy5.5 and CS/HHPC/Col I-GP-CurNPs(Cy5.5); whereas the fluorescence intensity in the CS/HHPC/Col I-GP-CurNPs(Cy5.5) group was significantly higher than that in the zein-150 MPa-P-Cur-Cy5.5 group (Figure 7A). With the extension of time, the fluorescence of DSS-induced UC mice treated with zein-150 MPa-P-Cur-Cy5.5 disappeared, whereas those treated with CS/HHPC/Col I-GP-CurNPs(Cy5.5) still showed a strong fluorescence signal at 24 h. The result indicated that the hydrogel shell of CS/HHPC/Col I-GP-CurNPs(Cy5.5) might prolong its retention time in the intestinal tract, thereby retarding intestinal clearance. However, since the abdominal fluorescence intensity could not accurately reflect the colonic targeting of CS/HHPC/Col I-GP-CurNPs(Cy5.5), further fluorescence analysis of organs was conducted by collecting visceral organs and intestinal tissues 24 h after administration (Figure 7B). Importantly, CS/HHPC/Col I-GP-CurNPs(Cy5.5) displayed great accumulation in inflamed colon tissue. The positive outcome was likely attributable to its hydrogel shell, which exhibited good tissue-adhesive properties and pH- and temperature-responsive characteristics, enabling it to endure harsh gastrointestinal conditions, achieve targeted adhesion to the colon, and be specifically degraded by colonic enzymes to release the CurNPs. The negatively charged CurNPs effectively maximized the polyvalent adhesive interaction between the CurNPs and the colon’s inflammation site, where positively charged transferrin is highly expressed, dramatically increasing their accumulation in the colon lesion and thus achieving precise and sustained release of Cur. Additionally, minimal fluorescence signals were observed in the liver and kidney in the CS/HHPC/Col I-GP-CurNPs(Cy5.5) group, indicating that the majority of the CS/HHPC/Col I-GP-CurNPs(Cy5.5) were excreted through the digestive system, with only a small fraction absorbed via the small intestine and subsequently distributed to the liver and kidney. These results demonstrated the colon-targeting properties and strong retention capacity of CS/HHPC/Col I-GP-CurNPs in DSS-induced UC mice.

### 2.17. Intervention with CS/HHPC/Col I-GP-CurNPs in DSS-Induced UC Mice

**Figure 8:**
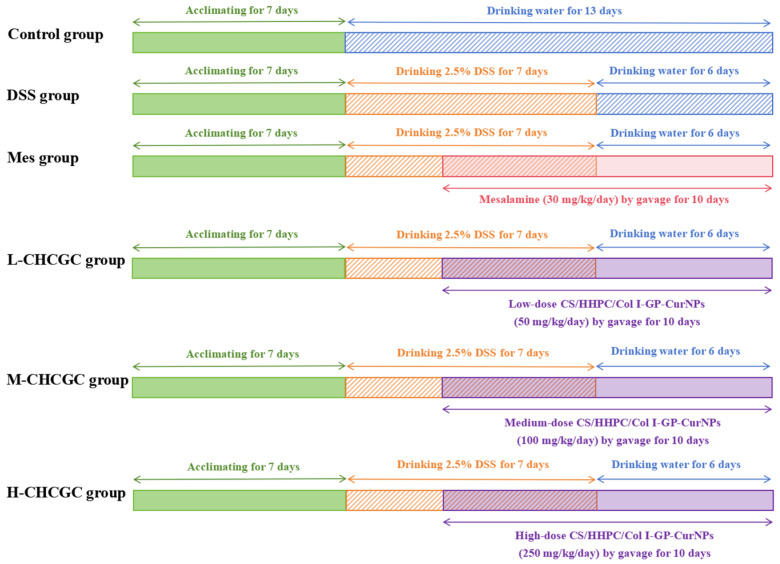
Animal experimental design.

**Figure 9:**
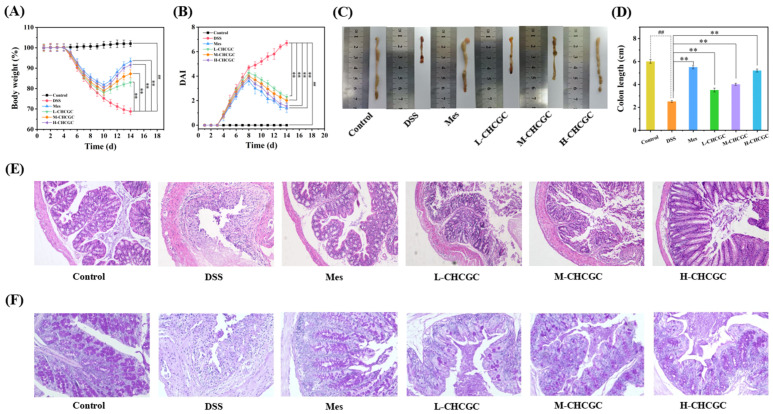
Treatment effects of CS/HHPC/Col I-GP-CurNPs in UC mice. Changes in (**A**) body weight, (**B**) DAI, (**C**) colon tissue photographs, and (**D**) colon length in UC mice following different treatments. (**E**) Representative H&E staining images and (**F**) PAS staining images of colon tissue (scale bar: 200 μm). Significance levels: ^##^ *p* < 0.01 vs. control group; ** *p* < 0.01 vs. LPS group.

Given the in vitro antioxidative and anti-inflammatory effects of CS/HHPC/Col I-GP-CurNPs, we further investigated their therapeutic effects in a DSS-induced UC mouse model. Mice were randomly assigned to six groups: control group, DSS group, positive control drug–mesalazine group (Mes group), low-dose CS/HHPC/Col I-GP-CurNPs group (L-CHCGC group), medium-dose CS/HHPC/Col I-GP-CurNPs group (M-CHCGC group), and high-dose CS/HHPC/Col I-GP-CurNPs group (H-CHCGC group). The experimental process is illustrated in Figure 8. The most common clinical indicators of UC are weight loss, colon shortening, and bloody stools [44]. Figure 9A,B show that mice in the control group displayed a slight weight gain and had DAI scores that were consistently zero, indicating normal growth [91]. DSS-treated mice exhibited significant weight loss from day 4 onward (*p* < 0.01), along with severe diarrhea and hematochezia, resulting in elevated DAI scores. On the final day of the experiment, the weight loss in the DSS group was 31.22 ± 2.05%, and the DAI score was 6.70 ± 0.43. In contrast, the CS/HHPC/Col I-GP-CurNPs intervention groups (L-CHCGC, M-CHCGC, and H-CHCGC) showed varying degrees of symptom inhibition, with the H-CHCGC group exhibiting the strongest effect. On the final day of the experiment, the weight loss in the H-CHCGC group was only 8.44 ± 1.39%, and the DAI score was 1.56 ± 0.28, comparable to that of the positive control Mes group. Colon morphology and length are important indicators of colitis severity [90]. Figure 9C,D show that the colons in the control group were uniform and smooth with no edema, whereas the colons in the DSS group exhibited edema, bleeding, and shortening [92]. Oral administration of CS/HHPC/Col I-GP-CurNPs alleviated colonic shortening, with colon lengths in the L-CHCGC, M-CHCGC, and H-CHCGC groups increasing by 0.40-fold, 0.60-fold, and 1.08-fold, respectively, compared to the DSS group. Notably, the H-CHCGC group had the longest colon length, similar to that of the control group, indicating that a high dose of CS/HHPC/Col I-GP-CurNPs provided a better therapeutic effect. Overall, these results suggested that CS/HHPC/Col I-GP-CurNPs effectively alleviated clinical symptoms in DSS-induced UC mice.

Evaluated pathological changes in colon tissue by examining H&E-stained sections (Figure 9E). Colon tissue from the control group showed an intact colonic mucosa, clear crypt structure, and densely packed goblet cells. Conversely, the DSS group exhibited extensive damage to the superficial epithelium and goblet cells, along with infiltration of inflammatory mononuclear leukocytes in the mucosal and submucosal layers, which are typical pathological manifestations of colitis [3]. Furthermore, edematous changes were observed at the interface between the intestinal mucosa and the muscular layers [93]. Notably, the L-CHCGC and M-CHCGC groups showed moderate recovery from pathological damage, with reduced inflammatory cell infiltration and less edema. The H-CHCGC and Mes groups showed the most significant alleviation, similar to that observed in healthy mice. Mucus-secreting goblet cells were restored to normal levels, intact crypts were formed, and inflammatory cell infiltration was eliminated. These collective findings suggested that CS/HHPC/Col I-GP-CurNPs effectively mitigated colon tissue inflammation.

The mucin content secreted by goblet cells in colon tissue was evaluated using PAS staining. Figure 9F indicates that the amount of mucin in the DSS group was significantly reduced compared to the control group, consistent with the observed loss of mucus-containing goblet cells in H&E-stained sections [94]. Intervention with H-CHCGC most effectively restored colonic mucin secretion, nearly reaching the level of the healthy control group. These findings suggested that the administration of CS/HHPC/Col I-GP-CurNPs strongly promoted the restoration of mucin secretion. These results resemble those from prior studies using Cur-loaded bilayer microgels to relieve colitis in mice, but it remains unclear whether mucin recovery was due to direct goblet cell regulation or secondary to anti-inflammatory effects [17]. In summary, these results indicated that CS/HHPC/Col I-GP-CurNPs effectively alleviated DSS-induced colitis.

### 2.18. CS/HHPC/Col I-GP-CurNPs Alleviated Inflammation, Modulated the Redox Balance, and Recovered Dysregulated Intestinal Barriers in DSS-Induced UC Mice

**Figure 10:**
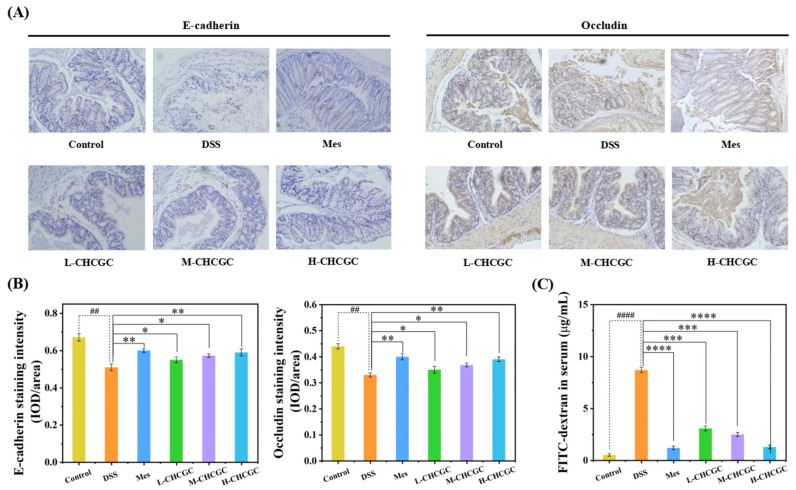
Intestinal barrier repair effects of CS/HHPC/Col I-GP-CurNPs in UC mice. (**A**) Images of colon tissue stained for E-cadherin and Occludin by immunohistochemistry following different treatments (scale bar: 200 μm) and (**B**) mean optical density of the colon sections. (**C**) Serum fluorescence intensity from different groups was measured 4 h after gavage with FITC-dextran. Significance levels: ^##^ *p* < 0.01 and ^####^ *p* < 0.0001 vs. control group; * *p* < 0.05, ** *p* < 0.01, *** *p* < 0.001, and **** *p* < 0.0001 vs. LPS group.

E-cadherin and Occludin, representative proteins of tight junction complexes, are crucial for maintaining intestinal barrier function and regulating permeability [11]. To determine the restorative effect of CS/HHPC/Col I-GP-CurNPs on the damaged intestinal barrier in UC-induced mice, immunohistochemistry was used to analyze the levels of E-cadherin and Occludin in mouse colons. Figure 10A,B show that the levels of E-cadherin and Occludin were markedly reduced in the DSS-treated group compared to the control group, indicating impaired intestinal barrier function (*p* < 0.01). CS/HHPC/Col I-GP-CurNPs treatment significantly upregulated these levels, indicating significant restoration of colonic barrier function (*p* < 0.05 and *p* < 0.01) [44]. Notably, the H-CHCGC group exhibited higher levels of E-cadherin and Occludin and a more intact intestinal epithelial barrier structure than the L-CHCGC and M-CHCGC groups, with effects comparable to those of the positive control drug Mes. This finding was supported by indirect evidence. Serum FITC-dextran levels indicate intestinal permeability; the higher the serum concentration, the greater the intestinal permeability [95]. Intestinal barrier integrity damage typically enhances intestinal permeability; Figure 10C shows that, compared to the DSS-treated group, UC mice treated with CS/HHPC/Col I-GP-CurNPs exhibited a significant, dose-dependent decrease in serum FITC-dextran concentration (*p* < 0.001 and *p* < 0.0001) [96]. These findings align with earlier studies on UC interventions [97,98], demonstrating that CS/HHPC/Col I-GP-CurNPs effectively reduced DSS-induced colon damage and played a significant role in restoring and preserving intestinal barrier function.

**Figure 11:**
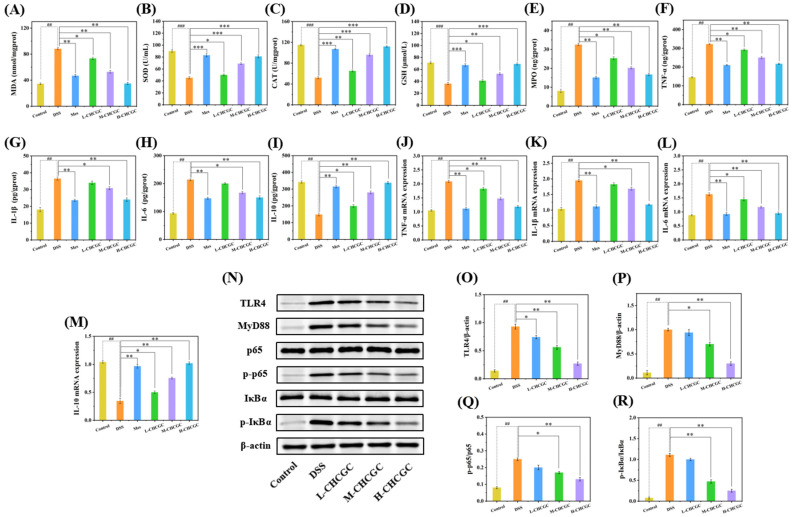
Anti-inflammatory and antioxidant activities of CS/HHPC/Col I-GP-CurNPs in UC mice. (**A**–**E**) Levels of MDA, SOD, CAT, GSH, and MPO in colon tissue following different treatments. (**F**–**I**) Levels of TNF-α, IL-1β, IL-6, and IL-10 in colon tissue following different treatments, and (**J**–**M**) their mRNA expression levels. (**N**) Western blotting images and (**O**–**R**) protein expression levels of TLR4, MyD88, p65, p-p65, IκBα, and p-IκBα in colon tissue following different treatments. Significance levels: ^##^ *p* < 0.01, and ^###^ *p* < 0.001 vs. control group; * *p* < 0.05, ** *p* < 0.01, and *** *p* < 0.001 vs. LPS group.

Oxidative stress is recognized as a vital pathological mechanism in UC and is typically followed by severe intestinal lesions. In this study, the levels of oxidative stress markers (MDA, SOD, CAT, and GSH) were analyzed to determine the therapeutic effect of CS/HHPC/Col I-GP-CurNPs on oxidative stress in UC-induced mice. Figure 11A–D illustrate that the levels of GSH, SOD, and CAT in DSS-treated mice decreased significantly compared to the control group (*p* < 0.001), along with a notable elevation in MDA levels (*p* < 0.01). However, the administration of L-CHCGC, M-CHCGC, and H-CHCGC significantly increased the levels of GSH, SOD, and CAT (*p* < 0.05, *p* < 0.01, and *p* < 0.001) and significantly decreased the level of MDA (*p* < 0.05 and *p* < 0.01), indicating that CS/HHPC/Col I-GP-CurNPs effectively alleviated intestinal oxidative stress injury and the symptoms of UC. In particular, the treatment effect in the H-CHCGC group was much greater than that in the other groups, nearly matching that of the healthy control group, indicating that high-dose CS/HHPC/Col I-GP-CurNPs have superior antioxidant properties. Activity of MPO, an endogenous enzyme produced by neutrophils that reflects the degree of inflammation and oxidative stress, was also analyzed [3]. Figure 11E shows that MPO activity was significantly higher in the DSS group than in the healthy control group (*p* < 0.01) and was significantly decreased by CS/HHPC/Col I-GP-CurNPs treatment (*p* < 0.05 and *p* < 0.01). In particular, the inhibitory effect of H-CHCGC was greater than that of L-CHCGC and M-CHCGC and showed no significant difference compared to the positive control drug Mes (*p* > 0.05). This indicated that CS/HHPC/Col I-GP-CurNPs reduced oxidative damage to intestinal epithelial cells at the injury site and decreased MPO levels in the inflamed colon, which is thought to indicate the inhibition of circulating inflammatory neutrophil recruitment and contribute to the redox balance recovery [99].

The local and systemic infiltration of inflammatory factors is a key pathological factor controlling UC initiation and progression [100]. Figure 11F–H show that the levels of the pro-inflammatory cytokines TNF-α, IL-1β, and IL-6 were remarkably increased in the DSS-treated group compared with the control group (*p* < 0.01), but were significantly decreased after treatment with CS/HHPC/Col I-GP-CurNPs (*p* < 0.05 and *p* < 0.01). Notably, the inhibitory effect of H-CHCGC on these pro-inflammatory cytokines was stronger than that of L-CHCGC and M-CHCGC and comparable to that of Mes. The level of the anti-inflammatory cytokine IL-10 showed a contrasting pattern (Figure 11I). The expression of these inflammatory cytokines at the transcriptional level in colon tissue was determined using RT-qPCR (Figure 11J–M). As expected, compared to the DSS group, the mRNA expression of pro-inflammatory cytokines TNF-α, IL-1β, and IL-6 decreased in a dose-dependent manner following treatment with CS/HHPC/Col I-GP-CurNPs, while the anti-inflammatory cytokine IL-10 increased in a dose-dependent manner; H-CHCGC demonstrated the greatest efficacy in suppressing pro-inflammatory cytokines and enhancing anti-inflammatory cytokine expression. These results confirmed that CS/HHPC/Col I-GP-CurNPs alleviated inflammation in DSS-induced colitis mice and were partly responsible for the amelioration of UC.

The TLR4/MyD88/NF-κB signaling pathway plays a central role in modulating inflammatory responses [44]. By recognizing pathogen- or damage-associated signals, this pathway triggers a cascade reaction that activates the NF-κB transcription factor and induces the release of numerous pro-inflammatory factors, resulting in inflammation and tissue injury characteristic of colitis [22]. We used Western blotting to examine key proteins—TLR4, MyD88, NF-κB, and IκBα—to assess the effect of CS/HHPC/Col I-GP-CurNPs on this pathway. As shown in Figure 11N–R, the DSS-induced group exhibited a notable upregulation in the expression of TLR4 and MyD88 proteins (*p* < 0.01), along with a significant increase in phosphorylated NF-κB (p-NF-κB) and IκBα (p-IκBα) (*p* < 0.01), indicating activation of the NF-κB signaling pathway [90]. In contrast, CS/HHPC/Col I-GP-CurNPs significantly downregulated these protein levels in a dose-dependent manner (*p* < 0.05 and *p* < 0.01). The results indicated that CS/HHPC/Col I-GP-CurNPs exerted anti-inflammatory effects by deactivating the TLR4/MyD88/NF-κB pathway. These results confirmed that CS/HHPC/Col I-GP-CurNPs markedly alleviated UC symptoms, reduced inflammation, modulated the redox balance, and effectively repaired intestinal mucosal barrier function.

### 2.19. Modulation of Gut Microbiota and Restoration of SCFA Production by CS/HHPC/Col I-GP-CurNPs in DSS-Induced UC Mice

Cur has been shown to modulate the intestinal microbiota and markedly improve UC [14]. However, because of the harsh digestive environment, free Cur struggles to reach the inflamed site in the colon. CS/HHPC/Col I-GP-CurNPs were proven to be capable of protecting Cur and delivering it to the colonic inflammatory sites, achieving its precise release; however, the impact of CS/HHPC/Col I-GP-CurNPs on the gut microbiota was unclear. Therefore, 16S rRNA gene high-throughput sequencing was employed to evaluate the impact of high doses of CS/HHPC/Col I-GP-CurNPs (H-CHCGC) on intestinal microbial homeostasis. Rarefaction and Shannon curves showed a progressive leveling off with increasing sample size, suggesting that the sequencing depth was sufficient to represent the gut microbiota composition (Supplementary Figure S4A,B) [88]. The α-diversity of gut microbiota in UC mice was measured to assess community diversity and richness (Supplementary Figure S4C). The DSS group exhibited a marked decrease in α-diversity compared to the healthy control group (*p* < 0.05 and *p* < 0.01). Treatment with H-CHCGC significantly enhanced α-diversity and increased intestinal microbial richness and diversity (*p* < 0.05 and *p* < 0.01). To assess β-diversity, principal coordinate analysis (PCoA) and non-metric multidimensional scaling (NMDS) were performed. These analyses demonstrated that the gut microbial profile in the DSS-treated mice markedly diverged from that of the control group, whereas the H-CHCGC treatment group exhibited a microbial profile closely resembling that of the control group (Supplementary Figure S4D,E) [91]. These findings suggested that oral administration of H-CHCGC could reverse β-diversity alterations of gut microbiota in UC mice and restore the structure of the gut microbiota. A Venn diagram illustrates the overlap of operational taxonomic units (OTUs) across the various treatment groups, revealing that 298 OTUs were commonly shared among them. Additionally, the numbers of unique OTUs in the control, DSS, and H-CHCGC groups were 1650, 1035, and 1347, respectively (Supplementary Figure S4F). These findings indicated that DSS administration markedly decreased species richness and diversity, which were subsequently recovered after treatment with H-CHCGC.

**Figure 12:**
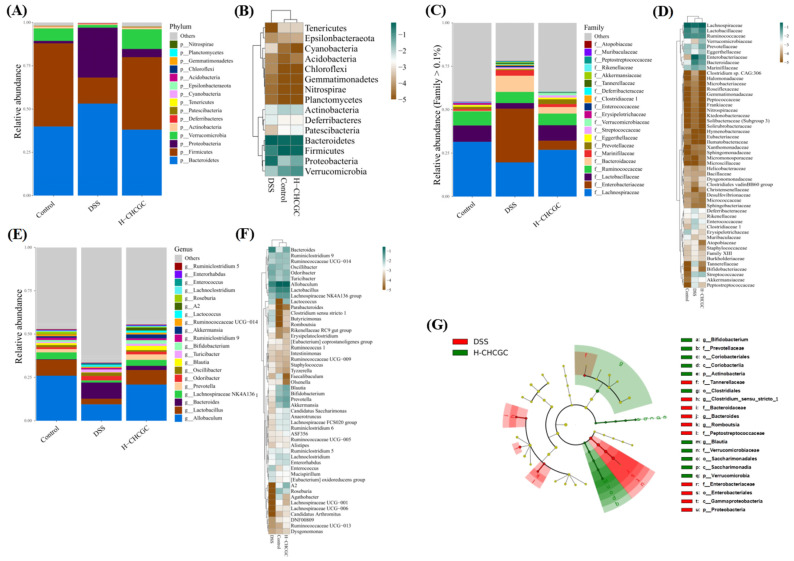
High-dose CS/HHPC/Col I-GP-CurNPs (H-CHCGC) modulated DSS-induced gut microbiota dysbiosis in UC mice. Composition and relative abundance of gut microbiota following different treatments at the phylum (**A**,**B**), family (**C**,**D**), and genus (**E**,**F**) levels, shown as histograms and heatmaps. (**G**) LEfSe analysis of the gut microbiota following different treatments.

Subsequently, the gut microbiota community composition was analyzed at the phylum, family, and genus levels across various groups. At the phylum level, *Firmicutes, Bacteroidetes, Proteobacteria,* and *Verrucomicrobia* were the dominant phyla, with *Firmicutes* and *Bacteroidetes* being the most abundant (Figure 12A). Previous studies have indicated that the *Firmicutes/Bacteroidetes* ratio is negatively correlated with colitis severity [90]. A comparative examination of gut microbial composition and relative abundance showed a marked decrease in the *Firmicutes* to *Bacteroidetes* ratio in the DSS-treated group, whereas administration of H-CHCGC effectively reversed this decline. Feng et al. similarly observed that Cur extract alleviated the reduced *Firmicutes/Bacteroidetes* ratio in the gut microbiota of DSS-induced colitis mice [92]. *Proteobacteria* have been proposed as a diagnostic marker for microbial imbalance in colitis [101]. *Verrucomicrobia* includes *Akkermansia*, which is associated with multiple health benefits. Compared to the control group, the relative abundance of *Proteobacteria* was increased, and that of *Verrucomicrobia* was reduced in the DSS-treated group. Notably, H-CHCGC attenuated the DSS-induced gut microbial changes at the phylum level by suppressing potential pathogenic bacteria and promoting beneficial bacteria, including reducing the abundance of *Proteobacteria* while enhancing that of *Verrucomicrobia*. The gut microbial heatmap at the phylum taxonomic level also showed that the control and H-CHCGC groups were tightly clustered and clearly distinguished from the DSS group, indicating that the gut microbiota composition in the H-CHCGC group was highly similar to that observed in healthy mice (Figure 12B).

At the family level, the main bacterial families identified were *Lactobacillaceae, Lachnospiraceae, Ruminococcaceae, Bacteroidaceae,* and *Enterobacteriaceae* (Figure 12C). *Lactobacillaceae* have been proven to offer multiple health benefits, including immunoregulation, anti-pathogenic activity, and strengthening of the epithelial barrier [102]. *Lachnospiraceae* metabolize various carbohydrates to produce butyric acid, which is associated with colitis remission [44]. *Ruminococcaceae* are closely related to intestinal health and affect nutritional metabolism, bile acid cycling, and disease development; the abundance of *Ruminococcaceae* is decreased in UC patients, which may be associated with disease pathogenesis [103]. *Bacteroidaceae* is closely associated with colitis and can induce colitis in susceptible animal models. In the DSS-induced colitis model, the abundance of *Bacteroidaceae* significantly increased; this increased abundance may exacerbate mucosal inflammatory responses, which is related to the aggravation of UC [104]. *Enterobacteriaceae* have a close relationship with colonic diseases; these bacteria can directly cause colonic diseases and indirectly affect colonic health by influencing the human immune response, antibiotic resistance, and the balance of the intestinal microbiota [105]. The relative abundance of beneficial bacteria—*Lactobacillaceae, Lachnospiraceae*, and *Ruminococcaceae*—was enriched in the healthy control group. DSS treatment markedly decreased the relative abundance of these beneficial bacteria while increasing that of harmful bacteria (*Bacteroidaceae* and *Enterobacteriaceae*), consistent with the gut microbial changes observed in UC patients [106]; however, H-CHCGC administration could restore the balance by increasing the relative abundance of beneficial bacteria and decreasing that of harmful bacteria. Similar patterns were observed in the gut microbial heatmap at the family taxonomic level (Figure 12D). Additionally, within the H-CHCGC group, *Prevotellaceae*—a beneficial bacterial family—showed significantly higher relative abundance than in the control and DSS groups. Specifically, *Prevotellaceae* produce high levels of SCFAs through carbohydrate metabolism, thereby exerting a relieving effect against intestinal inflammation [102]. These results showed that H-CHCGC effectively reversed the DSS-induced gut microbial alterations at the family level and demonstrated positive restoration of the dysregulated intestinal microecology.

Genus-level analysis showed that DSS significantly reduced the relative abundance of *Lactobacillus, Lachnospiraceae_NK4A136_group,* and *Allobaculum* (Figure 12E). *Lactobacillus* is widely acknowledged as a crucial probiotic that helps sustain intestinal homeostasis and has been shown to inhibit inflammation in UC patients [107]. It was reported that the *Lachnospiraceae_NK4A136_group* helps maintain intestinal health by promoting the function of intestinal epithelial cells through SCFA generation, and may positively counter intestinal diseases by regulating the gut microbiota structure [95]. *Allobaculum*, a prevalent gut symbiotic bacterium, produces butyric acid, which helps regulate the balance of the intestinal microbiota [108]. Moreover, in the DSS-induced group, the relative abundance of harmful bacteria such as *Bacteroides*—which produces extracellular toxins that can trigger intestinal inflammation—and *Turicibacter,* which is recognized for its positive correlation with the expression of proinflammatory cytokines, was significantly increased [109]. Importantly, H-CHCGC treatment notably increased the relative abundance of beneficial bacteria (*Lactobacillus, Lachnospiraceae NK4A136 group,* and *Allobaculum*) and decreased that of harmful ones (*Bacteroides* and *Turicibacter*). Next, the similarities and differences in the community composition across groups at the genus level were analyzed using a gut microbial heatmap (Figure 12F). The community composition of the H-CHCGC group was closest to that of the healthy control group. Notably, in the H-CHCGC group, the relative abundance of various beneficial bacteria, including *Bifidobacterium, Prevotella,* and *Blautia*, was significantly higher than that in the control and DSS groups. *Bifidobacterium* is widely recognized as a probiotic that coexists symbiotically in the animal gut; it helps mitigate UC by maintaining the mechanical barrier function of the intestinal tract, reducing pro-inflammatory cytokine secretion, and promoting SCFA production in collaboration with other beneficial bacteria [95]. Studies have shown that the relative abundance of *Prevotella* is higher in groups with a plant-based diet, suggesting that it may be a beneficial genus; additionally, *Prevotella* has a positive effect in alleviating UC symptoms [110]. *Blautia* is a novel functional genus with potential probiotic properties; it breaks down substances that the host fails to digest, thereby increasing the content of SCFAs in feces, biotransforms certain flavonoids, and produces secondary metabolites that inhibit harmful bacteria in the intestine and may help maintain an intestinal microecological balance by modulating immune responses [111]. An increase in *Akkermansia*, a beneficial bacteria that helps maintain intestinal barrier integrity, was also observed; its abundance shows a negative correlation with inflammation levels [107]. Taken together, these results emphasized that H-CHCGC administration increased the relative abundance of beneficial bacteria and reduced that of harmful bacteria, thereby effectively restoring DSS-induced dysregulation of intestinal microecology and alleviating UC.

Linear discriminant analysis effect size (LEfSe) was employed to further analyze gut microbiome differences between the DSS and H-CHCGC groups across various taxonomic levels. Figure 12G shows that the DSS group demonstrated a significant increase in the relative abundance of harmful bacteria—*Bacteroidaceae, Bacteroides, Enterobacteriaceae,* and *Proteobacteria*—all of which have been reported as detrimental bacteria that aggravate intestinal inflammation [44,104,105,109]. However, in the H-CHCGC group, various beneficial bacteria exerted a prominent effect, including *Prevotellaceae, Bifidobacterium,* and *Blautia* [95,102,111]. Overall, these findings indicated that H-CHCGC treatment could restore gut microbiota dysbiosis in UC mice.

SCFAs are key byproducts of dietary fiber fermentation by gut microbiota and are essential for intestinal homeostasis [88]. Supplementary Figure S5A–C show that levels of major SCFAs—such as acetic, propionic, and butyric acids—were significantly elevated following H-CHCGC administration compared to the DSS-induced group (*p* < 0.01). Butyric acid is especially prominent among SCFAs, as it is the primary energy source for intestinal epithelial cells, a modulator of intestinal barrier function, and demonstrates anti-inflammatory effects [44]. Other SCFAs, such as isobutyric acid, valeric acid, and isovaleric acid, showed similar patterns (Supplementary Figure S5D–F). As a result, the H-CHCGC intervention may increase SCFA levels, supporting the recovery of intestinal barrier function and reducing inflammatory responses in colitis.

## 3. Conclusions

In this study, an orally administrable spatiotemporally controlled nanohybrid polysaccharide-based hydrogel drug delivery system—namely CS/HHPC/Col I-GP-CurNPs—was successfully constructed for the colon-targeted release of Cur to alleviate UC. The delivery system demonstrated exceptional drug encapsulation efficiency, excellent biocompatibility, and well-controlled drug release properties. Particularly, its polysaccharide-based hydrogel shell (CS/HHPC/Col I-GP) exhibited good mechanical strength and swelling performance, tissue-adhesive properties, and pH- and temperature-responsive characteristics, enabling it to endure harsh gastrointestinal conditions, achieve targeted adhesion to the colon, and be specifically degraded by colonic enzymes. The CurNPs released in the colon benefit from their negatively charged characteristics, enabling accumulation at the positively charged inflamed sites and achieving precise and sustained release of Cur. Most importantly, CS/HHPC/Col I-GP-CurNPs exhibited satisfactory UC treatment efficacy through a multifaceted mechanism. On the one hand, CS/HHPC/Col I-GP-CurNPs repolarized M1 macrophages into the M2 phenotype, thereby transitioning the pro-inflammatory environment into an anti-inflammatory state and consequently exerting anti-inflammatory and protective effects. On the other hand, CS/HHPC/Col I-GP-CurNPs downregulated oxidative stress and inflammatory cytokine production to alleviate UC by deactivating the TLR4/MyD88/NF-κB pathway. Moreover, CS/HHPC/Col I-GP-CurNPs effectively restored intestinal mucosal barrier integrity and function, improved gut microbiota richness and diversity, and thereby normalized the gut microenvironment. To conclude, this advanced nanohybrid polysaccharide-based hydrogel oral drug delivery system demonstrates great potential for UC treatment.

## 4. Materials and Methods

### 4.1. Materials

CS (Mw: 300 kDa, DDA = 85%), GP (C_3_H_7_Na_2_O_6_P·5H_2_O), HPC (Mw: 480 kDa, DS = 1.26, MS = 1.88), and acetonitrile (chromatographic grade) were obtained from Sigma (USA). LPS, 4′,6-diamidino-2-phenylindole (DAPI), Dulbecco’s minimum essential medium (DMEM), fetal bovine serum (FBS), and Coumarin-6 (Cou6) were obtained from Thermo Fisher Scientific Co., Ltd. (Shanghai, China). CD86 rabbit mAb, anti-CD206 antibody, TLR4 rabbit pAb, MyD88 rabbit pAb, NF-κB p65/RelA rabbit mAb, phospho-NF-κB p65/RelA-S536 rabbit pAb, IκBα rabbit mAb, and phospho-IκBα-S32 rabbit mAb were obtained from Servicebio (Wuhan, China). Biochemical and enzyme-linked immunosorbent assay (ELISA) kits were obtained from Jiangsu Enzyme Immuno Industry Co., Ltd. (Yancheng, China).

### 4.2. Preparation and Characterization of the Cur-Loaded Nanoparticles

The Cur-loaded NPs (zein-150 MPa-P-Cur), consisting of high hydrostatic pressure-treated zein and pectin, were prepared using an antisolvent precipitation method. For detailed preparation methods, formation mechanisms, structural characteristics, stability, and bioaccessibility of the Cur-loaded NPs, please refer to a recent report from our laboratory [43].

### 4.3. Preparation of the Dual-Responsive Polysaccharide-Based Hydrogels

Synthesized following a previously described procedure [35].

Solution 1: Dissolve CS in 1% (*w*/*v*) acetic acid to obtain a 3.33% (*w*/*v*) CS solution. Subsequently, incorporate Col I into the CS solution to yield a final concentration of 16.7 mg/mL.

Solution 2: NaHCO_3_ and Na_2_CO_3_ were added to the GP solution (0.3 g/mL) to yield final concentrations of 0.1 g/mL and 5 mg/mL, respectively.

Solution 3: HPC was added to deionized water at 60 °C to produce both a low-concentration HPC solution (LHPC, 16.5 mg/mL) and a high-concentration HPC solution (HHPC, 33 mg/mL).

In the four-phase hydrogel system, the volume ratio of solution 1 to solution 2 to solution 3 was set at 6:2:2, with formulations summarized in Table 2. All hydrogel preparations were carried out in an ice bath, followed by storage at 4 °C.

### 4.4. Characterization of the Dual-Responsive Polysaccharide-Based Hydrogels

#### 4.4.1. Gelation Time

A gel solution in a test tube was maintained at 4 °C and then transferred to a 37 °C water bath [112]. Gelation time was measured by observing when the gel solution ceased to flow upon inversion of the test tube.

#### 4.4.2. Determination of Porosity

According to the liquid displacement technique, the dried hydrogels were immersed in absolute alcohol, followed by the removal of excess alcohol using filter paper [48]. The calculation was based on Equation (1):

(1) $$Porosity\;(\%)=(M_{2}-M_{1})/\rho V\times 100$$

where *ρ* and *V* are the density and volume of absolute ethanol, respectively, and *M*_1_ and *M*_2_ are the weights prior to and following immersion in absolute ethanol.

#### 4.4.3. Determination of Mechanical Strength

The gel solution was fully gelatinized, and its mechanical properties were subsequently evaluated using a Q800 thermomechanical analyzer (TA Instruments, Ltd., New Castle, DE, USA) at frequencies of 1, 2, and 5 Hz.

#### 4.4.4. FTIR

FTIR spectra of the individual components and hydrogels were recorded using an FTIR spectrometer (Nicolet iS10 FTIR, Thermo Fisher Scientific, Waltham, MA, USA).

#### 4.4.5. SEM

Microstructures of the hydrogels were examined by SEM (Hitachi SU8010, Tokyo, Japan), and images with a magnification of 180× were obtained at 5 kV.

#### 4.4.6. Rheological Properties

The rheological behavior of the hydrogels was determined using a rotational rheometer (TA DHR-3, New Castle, DE, USA) [24]. The storage modulus (G′) and loss modulus (G″) were measured as functions of strain (0.01–1.2%) and frequency (0.01–10 Hz) at 37 °C.

#### 4.4.7. Swelling Behavior

The swelling rate of the hydrogels was assessed by direct weighing [25]. The dried hydrogels were weighed to determine their initial mass (*W*_0_) and then immersed in PBS buffer (pH 7.4, 37 °C) with gentle agitation. At specified time intervals, the samples were removed, weighed, and recorded as *W_t_*. The calculation was based on Equation (2):

(2) $$Swelling\;rate\;(\%)=(W_{t}-W_{0})/W_{0}\times 100$$

#### 4.4.8. Biodegradation Behavior

The biodegradation of the hydrogels was assessed through simulation of the gastrointestinal environment [3]. Simulated gastric fluid (SGF, pH 2.2) contained pepsin in a dilute hydrochloric acid solution; simulated intestinal fluid (SIF, pH 6.8) included trypsin in PBS buffer; and simulated colonic fluid (SCF, pH 7.4) consisted of a PBS buffer incorporating extracellular enzymes. The hydrogels were placed in SGF, SIF, or SCF and incubated in a thermostatic shaker at 37 °C. The samples were removed and weighed at specific time intervals. The calculation was based on Equation (3):

(3) $$Degradation\;rate\;(\%)=(W_{s}-W_{r})/W_{s}\times 100$$

where *W_s_* and *W_r_* are the weights of the initial hydrogels and the degraded hydrogels, respectively.

#### 4.4.9. Viscosity and Adhesion

##### Physical Adhesion Test

Adhesion of the hydrogels was measured by observing a ball sliding down a slope inclined at a 45° angle. The bottom surface was covered with a hydrogel layer. The distance the ball traveled on the hydrogel-covered surface was recorded.

##### Tissue Residue Test

A 10 mL aliquot of hydrogel was applied to the pig’s colon, followed by rinsing with 10 mL of distilled water. The volume of the liquid that flowed out was recorded as *V*. The volume of hydrogel remaining attached to the colon was calculated as (20 − *V*).

##### Adhesion Force Test

Spread the gel solution onto a piece of colon tissue, and then apply another piece of tissue onto the surface of the gel solution by gently pressing. After gelation, the sample was subjected to tearing using a mechanical testing machine to determine the peak force necessary.

### 4.5. Preparation of the Dual-Responsive Polysaccharide-Based Hydrogels Loaded with Cur Nanoparticles

The preparation process of the dual-responsive polysaccharide-based hydrogels loaded with CurNPs refers to a previously described physical pre-formulation method [48,113]. The Cur-loaded NPs (zein-150 MPa-P-Cur) were prepared using an antisolvent precipitation method. In the second phase, a suspension of Cur-loaded NPs (1 wt.% Cur) was incorporated into the hydrogel precursor solution under stirring. The dual-responsive polysaccharide-based hydrogels loaded with CurNPs were designated as CS-GP-CurNPs, CS/LHPC-GP-CurNPs, CS/HHPC-GP-CurNPs, CS/Col I-GP-CurNPs, CS/LHPC/Col I-GP-CurNPs, and CS/HHPC/Col I-GP-CurNPs according to their composition.

### 4.6. Characterization of the Dual-Responsive Polysaccharide-Based Hydrogels Loaded with Cur Nanoparticles

#### 4.6.1. Encapsulation Efficiency

The encapsulation efficiency of Cur in the hydrogels loaded with CurNPs was assessed using high-performance liquid chromatography (HPLC) (EClassical 3200, Yilite, Dalian, China), following a previously reported protocol [12]. The calculation was based on Equation (4):

(4) $$\mathrm{Encapsulation}\;\mathrm{efficiency}\;(\%)=(M_{0}-M_{1})/M_{0}\times 100$$

where *M*_0_ is the total weight of Cur used to prepare the hydrogels loaded with CurNPs, and *M*_1_ is the weight of Cur in the supernatant.

#### 4.6.2. Zeta Potential

The hydrogels loaded with CurNPs were diluted and analyzed for zeta potential using a Malvern Zetasizer Nano ZS90 (Malvern Panalytical, Worcestershire, UK) [114].

#### 4.6.3. Drug Release Under Different pH Environments and Temperatures

The pH-responsive drug release behavior of the hydrogels loaded with CurNPs was investigated in solutions at a temperature of 37 °C under pH 7.4 and pH 2.2 conditions. The temperature-responsive drug release behavior of the hydrogels loaded with CurNPs was evaluated in pH 7.4 solutions at 37 °C and 25 °C [80]. The samples were incubated in the corresponding buffer solutions at the appropriate temperatures. At specific time intervals, the buffer containing Cur was collected, and the Cur concentration was measured following the procedure outlined in Section 4.6.1. The calculation was based on Equation (5):

(5) $$Cumulative\;release\;(\%)=\left[C_{i}V_{i}+\overset{n}{\underset{i=1}{\sum }}(C_{i-1}V_{s})\right]/m_{t}\times 100$$

where *C_i_* is the Cur concentration released at time *t*, *V_i_* is the volume of the release medium, *V_s_* is the volume removed, and *m_t_* is the initial mass of Cur encapsulated within the CurNPs incorporated into the hydrogel.

#### 4.6.4. In Vitro Drug Release

To assess the sensitivity of the hydrogels loaded with CurNPs to both pH and colon-specific enzymes, a release study was conducted in SGF, SIF, and SCF containing the corresponding enzymes using a dynamic dialysis method [3]. The samples were incubated in each fluid at 37 °C with continuous agitation. At specified intervals, aliquots were collected, centrifuged to obtain the supernatant, and filtered to determine the Cur concentration by HPLC using the procedure outlined in Section 4.6.1. The cumulative release of Cur was calculated using Equation (5).

The release kinetics and mechanism of Cur were analyzed using the zero-order, first-order, Higuchi, and Korsmeyer–Peppas models. The fitting parameters are presented in Supplementary Table S1.

### 4.7. Cell Experiments

#### 4.7.1. Cell Cytotoxicity Assay

The viability of LPS-stimulated and CS/HHPC/Col I-GP-CurNPs-treated RAW264.7 cells was evaluated using a CCK-8 kit. Cells were seeded in 96-well plates and treated with LPS (0.25–8 μg/mL) or CS/HHPC/Col I-GP-CurNPs (1–100 μg/mL) for 24 h. After adding the CCK-8 solution and incubating for 2 h, absorbance was measured at 450 nm using a microplate reader (BioRad, Hercules, CA, USA). Cell inflammation was induced by stimulation with 1 µg/mL LPS. Finally, appropriate concentrations of LPS and CS/HHPC/Col I-GP-CurNPs were selected for use in the following experiments. The calculation was based on Equation (6):

(6) $$Cell\;viability\;(\%)=(A_{s}-A_{b})/A_{c}-A_{b}\times 100$$

where *A_s_*, *A_c_*, and *A_b_* are the absorbances of the sample well, the control well, and the blank well, respectively.

#### 4.7.2. Cellular Uptake Assay

Cou6-labeled CurNPs were encapsulated in the hydrogel carrier matrix to obtain CS/HHPC/Col I-GP-Cou6NPs for evaluating cellular uptake efficiency. RAW264.7 cells were incubated in 6-well plates and then replaced with fresh DMEM containing CS/HHPC/Col I-GP-Cou6NPs (1, 5, and 10 μg/mL). The medium was removed at specific time points, and the cells were rinsed, fixed with 4% paraformaldehyde, permeabilized, and incubated with anti-rhLF followed by anti-rabbit IgG. Rhodamine B-labeled phalloidin and DAPI were used to counterstain the cytoskeleton and nuclei, respectively, and cells were observed using a fluorescence microscope (Olympus, Tokyo, Japan). Cellular uptake efficiency of CS/HHPC/Col I-GP-Cou6NPs was quantified by flow cytometry (FCM) (Beckman Coulter, Indianapolis, IN, USA).

#### 4.7.3. Cell Scratch Assay

RAW264.7 cells were cultured in 6-well plates until confluent, and a scratch was made using a pipette tip to simulate damage. The cells displaced by the scratch were removed by washing. After adding medium containing 1.5% FBS to each well, the cells were treated with CS/HHPC/Col I-GP (1, 5, and 10 μg/mL) or CS/HHPC/Col I-GP-CurNPs (1, 5, and 10 μg/mL). The scratch width was examined at specified time points using an inverted microscope (Olympus IX73, Tokyo, Japan) and quantified with ImageJ.

#### 4.7.4. Cellular Antioxidant and Anti-Inflammatory Effect Evaluation

RAW264.7 cells were cultured in 6-well plates and treated with CS/HHPC/Col I-GP-CurNPs (1, 5, and 10 μg/mL) for 24 h, followed by replacement with fresh medium containing LPS (1 μg/mL) for another 24 h to stimulate the cells. The levels of total antioxidant capacity (T-AOC), total superoxide dismutase (T-SOD), catalase (CAT), NO, malondialdehyde (MDA), glutathione (GSH), and inducible nitric oxide synthase (iNOS) were measured using biochemical assay kits. The concentrations of the proinflammatory cytokines tumor necrosis factor (TNF)-α, interleukin (IL)-6, IL-1β, IL-18, IL-23, and the anti-inflammatory cytokine IL-10 were quantified using the corresponding ELISA kits.

#### 4.7.5. Macrophage Phenotypic Transformation

RAW264.7 cells were cultured in 24-well plates. Following this, the cells were stimulated with LPS (1 μg/mL) and treated with CS/HHPC/Col I-GP-CurNPs (10 μg/mL) for 24 h. After removal of the medium, the cells were washed and fixed with 4% paraformaldehyde. After permeabilization and blocking, the cells were incubated with CD86 rabbit mAb and anti-CD206 antibody, followed by staining with cyanine 3-labeled goat anti-rabbit IgG secondary antibody and DAPI for nuclear visualization. The cells were observed using a fluorescence microscope (Discover Echo, RVL-100, San Diego, CA, USA).

Western blotting was performed to detect CD86, CD206, and β-actin in cells. Images were captured with a Bio-Rad imaging system (Hercules, CA, USA) and subsequently quantified using ImageJ 9.1 software.

### 4.8. Animal Experiments

#### 4.8.1. Hemolytic Assay

According to a previously described method [115], hemolysis assays were performed to assess the hemocompatibility of CS/HHPC/Col I-GP-CurNPs. The samples were categorized into positive control (Triton-X-100), negative control (PBS), and CS/HHPC/Col I-GP-CurNPs (0.125–2 mg/mL) groups. The calculation was based on Equation (7):

(7) $$Hemolysis\;rate\;(\%)=(O_{A}-O_{D})/(O_{B}-O_{D})\times 100$$

where *O_A_*, *O_B_*, and *O_D_* are the absorbance readings of the sample, positive control, and negative control, respectively.

#### 4.8.2. Construction and Treatment of a DSS-Induced UC Mouse Model

Animal studies were authorized by the Ethics Committee on Laboratory Animal Welfare of Weishi Testing Technology Service Co., Ltd. (Changchun, China; Animal No. 20241026-01). Forty-eight 8-week-old male C57BL/6 mice were randomly assigned to six groups: control group, DSS group, positive control drug–mesalazine group (Mes group), low-dose CS/HHPC/Col I-GP-CurNPs group (L-CHCGC group), medium-dose CS/HHPC/Col I-GP-CurNPs group (M-CHCGC group), and high-dose CS/HHPC/Col I-GP-CurNPs group (H-CHCGC group). Figure 8 illustrates the experimental design. Body weight and disease activity index (DAI) were measured to assess colitis severity. Feces were collected before sacrifice, followed by the collection of colon tissues.

#### 4.8.3. In Vivo Imaging

To evaluate the ability of CS/HHPC/Col I-GP-CurNPs to target the inflamed colon, CurNPs were labeled with the Cy5.5 fluorescent dye and then incorporated into the hydrogel matrix, yielding CS/HHPC/Col I-GP-CurNPs(Cy5.5). DSS-induced UC mice were orally administered Cy5.5-labeled CurNPs (zein-150 MPa-P-Cur-Cy5.5) and CS/HHPC/Col I-GP-CurNPs(Cy5.5) at an equivalent Cy5.5 dose of 4 mg/kg. Fluorescence images were acquired at specific time intervals using an in vivo imaging system (IVIS) (PerkinElmer, Shelton, CT, USA) after the mice were anesthetized. At 24 h post-administration, the mice were humanely euthanized, and major organs—such as the heart, liver, spleen, lungs, kidneys, and intestines—were harvested for fluorescence imaging analysis using the IVIS system.

#### 4.8.4. Histopathological and Immunohistochemical Analysis

The prepared colon tissue sections were stained with hematoxylin and eosin (H&E) and periodic acid–Schiff (PAS) [96], followed by analysis under an optical microscope (Nikon Co., Tokyo, Japan). The expression of Occludin and E-cadherin in colon tissue was evaluated using immunohistochemistry, and the protein levels were estimated by measuring the average optical density with Image-Pro Plus 6.0.

#### 4.8.5. In Vivo Intestinal Permeability Analysis

Mice received 500 mg/kg body weight of fluorescein 5(6)-isothiocyanate (FITC)-dextran orally to assess intestinal permeability [91]. Serum was collected 4 h after gavage, and FITC fluorescence intensity was measured using a multifunctional spectrophotometer (Varioskan, Thermo Fisher Scientific Co., Waltham, MA, USA) with emission at 520 nm.

#### 4.8.6. Evaluation of In Vivo Anti-Oxidative Stress and Anti-Inflammatory Activity

The levels of CAT, SOD, GSH, MDA, and myeloperoxidase (MPO) in the colon tissue supernatant were determined using biochemical assay kits. Inflammatory cytokines (TNF-α, IL-1β, IL-6, and IL-10) were assessed using ELISA kits.

#### 4.8.7. Real-Time Fluorescent Quantitative Polymerase Chain Reaction (RT-qPCR) Analysis

Total RNA was isolated from colon tissue using TRIzol reagent. A reverse transcription kit was used to perform first-strand cDNA synthesis. Then, RT-qPCR was used to assess the mRNA expression levels of the target genes of interest. The primers are listed in Supplementary Table S2.

#### 4.8.8. Western Blot Analysis

Western blotting for TLR4, MyD88, NF-κB p65, p-NF-κB p65, IκBα, p-IκBα, and β-actin in colon tissue was performed. Images were captured using a Bio-Rad imaging system (Hercules, CA, USA) and subsequently quantified using ImageJ software.

#### 4.8.9. Gut Microbiota and SCFA Content Analysis

Feces from the control, DSS, and H-CHCGC groups were collected before the mice were sacrificed. Gut microbiota were analyzed using 16S rRNA sequencing technology targeting the V3–V4 region. Sample processing was completed at Pinosen Biotechnology Co., Ltd. (Shanghai, China), and the analysis was performed on a cloud platform. The cecal contents of these three groups were analyzed using a gas chromatograph (GC9790II MC-02, Meruochen, Shenzhen, China).

### 4.9. Statistical Analysis

All experimental data were obtained in triplicate and are expressed as mean ± standard deviation (SD). Group significance analysis was assessed using the two-sample *t*-test and one-way ANOVA.

## Figures and Tables

**Table 1:** Release kinetics analysis of the dual-responsive polysaccharide-based hydrogels loaded with CurNPs.

Sample	Kinetic Model				
	Zero-Order	First-Order	Higuchi	Korsmeyer–Peppas	
	R^2^	R^2^	R^2^	R^2^	n
CS-GP-CurNPs	0.6754	0.7712	0.8356	0.9976	0.2213
CS/LHPC-GP-CurNPs	0.4789	0.7145	0.8726	0.9934	0.2925
CS/HHPC-GP-CurNPs	0.7854	0.6668	0.8189	0.9812	0.3475
CS/Col I-GP-CurNPs	0.4490	0.6725	0.9143	0.9910	0.2344
CS/LHPC/Col I-GP-CurNPs	0.5632	0.6883	0.8123	0.9889	0.3051
CS/HHPC/Col I-GP-CurNPs	0.6332	0.6915	0.9031	0.9766	0.3383

**Table 2:** Composition of the hydrogels.

Samples	CS (*w*/*v*)%	GP (*w*/*v*)%	HPC (*w*/*v*)%	Col I (mg/mL)
CS-GP	2	6	-	-
CS/LHPC-GP	2	6	0.33	-
CS/HHPC-GP	2	6	0.67	-
CS/Col I-GP	2	6	-	10
CS/LHPC/Col I-GP	2	6	0.33	10
CS/HHPC/Col I-GP	2	6	0.67	10

## Data Availability

The original contributions presented in this study are included in the article/Supplementary Material. Further inquiries can be directed to the corresponding authors.

## Supplementary Materials

The following supporting information can be downloaded at: https://www.mdpi.com/article/10.3390/gels12060503/s1, Figure S1: (A) Biodegradation behavior of different dual-responsive polysaccharide-based hydrogels in SGF, SIF, and SCF. (B) Physical adhesion, (C) amounts adhered to the colon tissue surface, and (D) adhesion force to the colon tissue surface after gelation of different dual-responsive polysaccharide-based hydrogels; Figure S2: RAW264.7 cell viability after treatment with (A) LPS and (B) CS/HHPC/Col I-GP-CurNPs at different concentrations; Figure S3: Hemolysis images and hemolytic rates of CS/HHPC/Col I-GP-CurNPs at different concentrations incubated with plasma for 4 h; Figure S4: High-dose CS/HHPC/Col I-GP-CurNPs (H-CHCGC) modulated DSS-induced gut microbiota dysbiosis in UC mice; Figure S5: SCFAs metabolite levels in cecal contents were determined by gas chromatography following different treatments; Table S1: Release kinetic models and equations; Table S2: Names and sequences of primers used for RT-qPCR.

## Abbreviations

The following abbreviations are used in this manuscript:

UC	Ulcerative colitis
GIT	Gastrointestinal tract
CS	Chitosan
HPC	Hydroxypropyl cellulose
Col I	Collagen type I
GP	β-glycerol phosphate disodium salt pentahydrate
FDA	Food and Drug Administration
CurNPs	Curcumin nanoparticles
NO	Nitric oxide
DSS	Dextran sulfate sodium
LPS	Lipopolysaccharide
