# Supplementary material for: Advanced Targeted Curcumin Delivery Using Spatiotemporally Controlled Nanohybrid Polysaccharide-Based Hydrogel for Ulcerative Colitis Therapy
Source: Gels. 2026 Jun 5;12(6):503. doi: 10.3390/gels12060503 (PMC13297880; doi:10.3390/gels12060503)
Supplement: Supplementary file 1 [file gels-12-00503-s001.zip › gels-4259965-supplementary.pdf]

Advanced targeted curcumin delivery using spatiotemporally controlled nanohybrid polysaccharide-based hydrogel for ulcerative colitis therapy

Nan Wang[a,b], Tingting Liu[a,c,*]

[a] School of Food Science and Engineering, Jilin Agricultural University, Changchun 130118, China

[b] Key Laboratory of Technological Innovations for Grain Deep-processing and High-effeciency Utilization of By-products of Jilin Province, Changchun 130118, China

[c] Engineering Research Center of Grain Deep-processing and High-effeciency Utilization of Jilin Province, Changchun 130118, China

[*]**Corresponding author:** Tingting Liu, School of Food Science and Engineering, Jilin Agricultural University, Changchun, 130118 China.

Email: Liutingting@jlau.edu.cn

Tel: +0431-8453295

Other authors emails:

Nan Wang (First author): 18943920137@163.com

Supplementary Table S1

Release kinetic models and equations.

Kinetic model	Equation
Zero-order	$M_t / M_\infty = K_0 t$
First-order	$M_t / M_\infty = 100 \times [1 - \exp(K_1 t)]$
Higuchi	$M_t / M_\infty = K_h t^{1/2}$
Korsmeyer–Peppas	$M_t / M_\infty = K_p t^n$

Note: M_t and M_∞ are the amount of drug released at time t and equilibrium, respectively; K_0, K_1, K_h, and K_p are the kinetic constants; and n is the release exponent that characterizes the drug release mechanism. For values of $n < 0.5$, the drug is released from the polymeric matrix into the release medium following Fickian diffusion; if $n = 1$, the mechanism is considered case II transport; if $0.5 < n < 1$, release from the polymeric matrix is referred to as non-Fickian diffusion.

Supplementary Table S2

Names and sequences of primers used for RT-qPCR.

Genes	Forward primer	Reverse primer
TNF-α	CAGGTCTATTTTGGGATCATTGCC	TCCCTGATTTCTAAGTGTTGCTGT
IL-1β	CTCCATGAGCTTTGTACAAGG	TGCTGATGTACCAGTTGGGG
IL-6	CCCCAATTTCCAATGCTCTCC	CGCACTAGGTTTGCCGAGTA
IL-10	TTCTTTCAAACAAAGGACCAGC	GCAACCCAAGTAACCCTTAAAG
GAPDH	AGGAGCGAGACCCCACTAACATCAA	ATGGGGGCATCGGCAGAAGGGGCGG

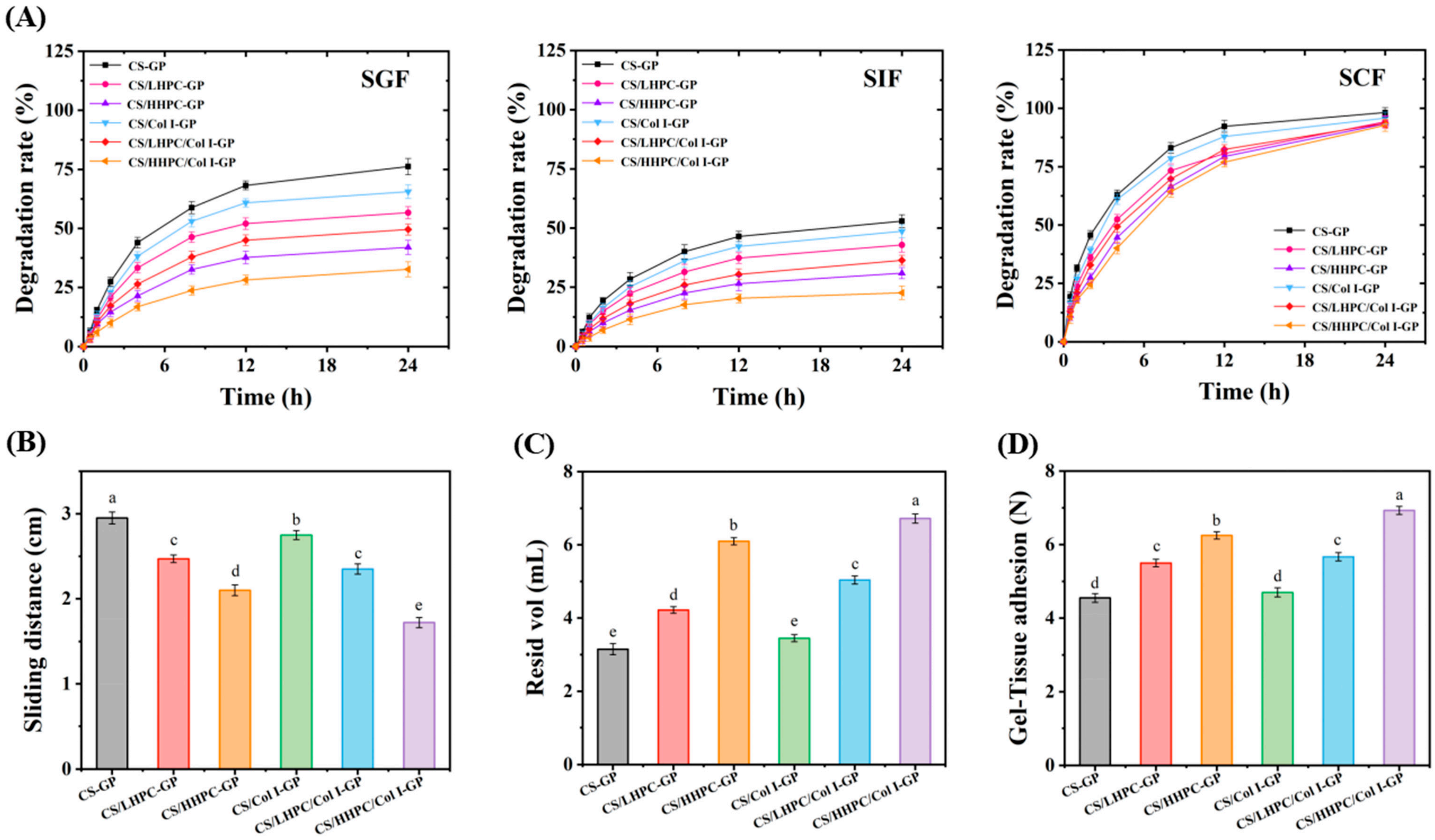

Supplementary Figure S1. (A) Biodegradation behavior of different dual-responsive polysaccharide-based hydrogels in SGF, SIF, and SCF. (B) Physical adhesion, (C) amounts adhered to the colon tissue surface, and (D) adhesion force to the colon tissue surface after gelation of different dual-responsive polysaccharide-based hydrogels. Significant differences in values ($p < 0.05$) are denoted by distinct letters (a–e).

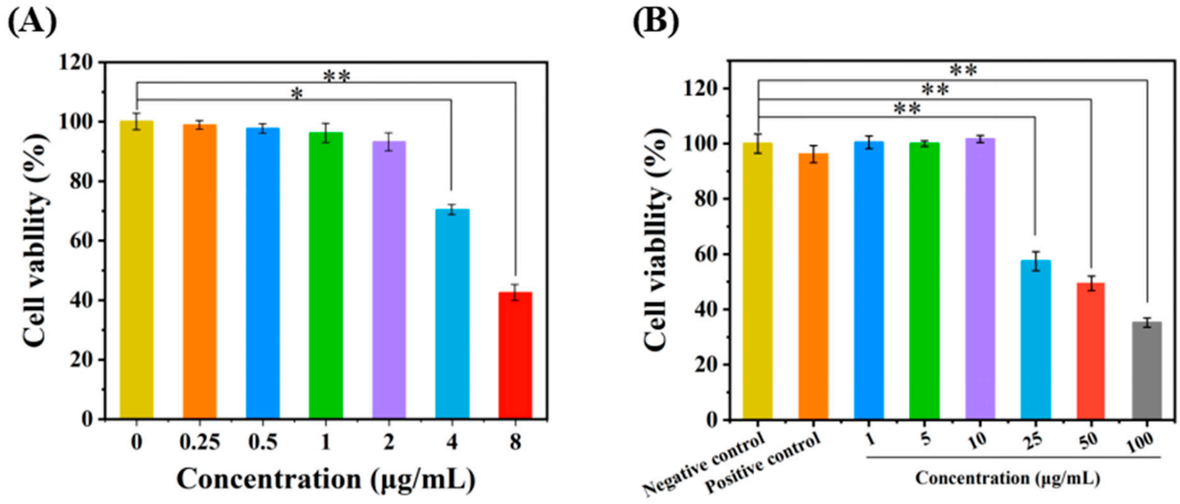

Supplementary Figure S2. RAW264.7 cell viability after treatment with (A) LPS and (B) CS/HHPC/Col I-GP-CurNPs at different concentrations. Significance levels: *$p < 0.05$ and **$p < 0.01$.

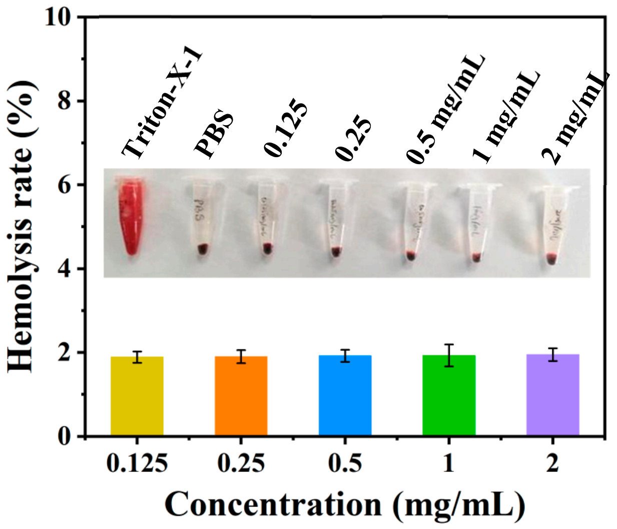

Supplementary Figure S3. Hemolysis images and hemolytic rates of CS/HHPC/Col I-GP-CurNPs at different concentrations incubated with plasma for 4 h.

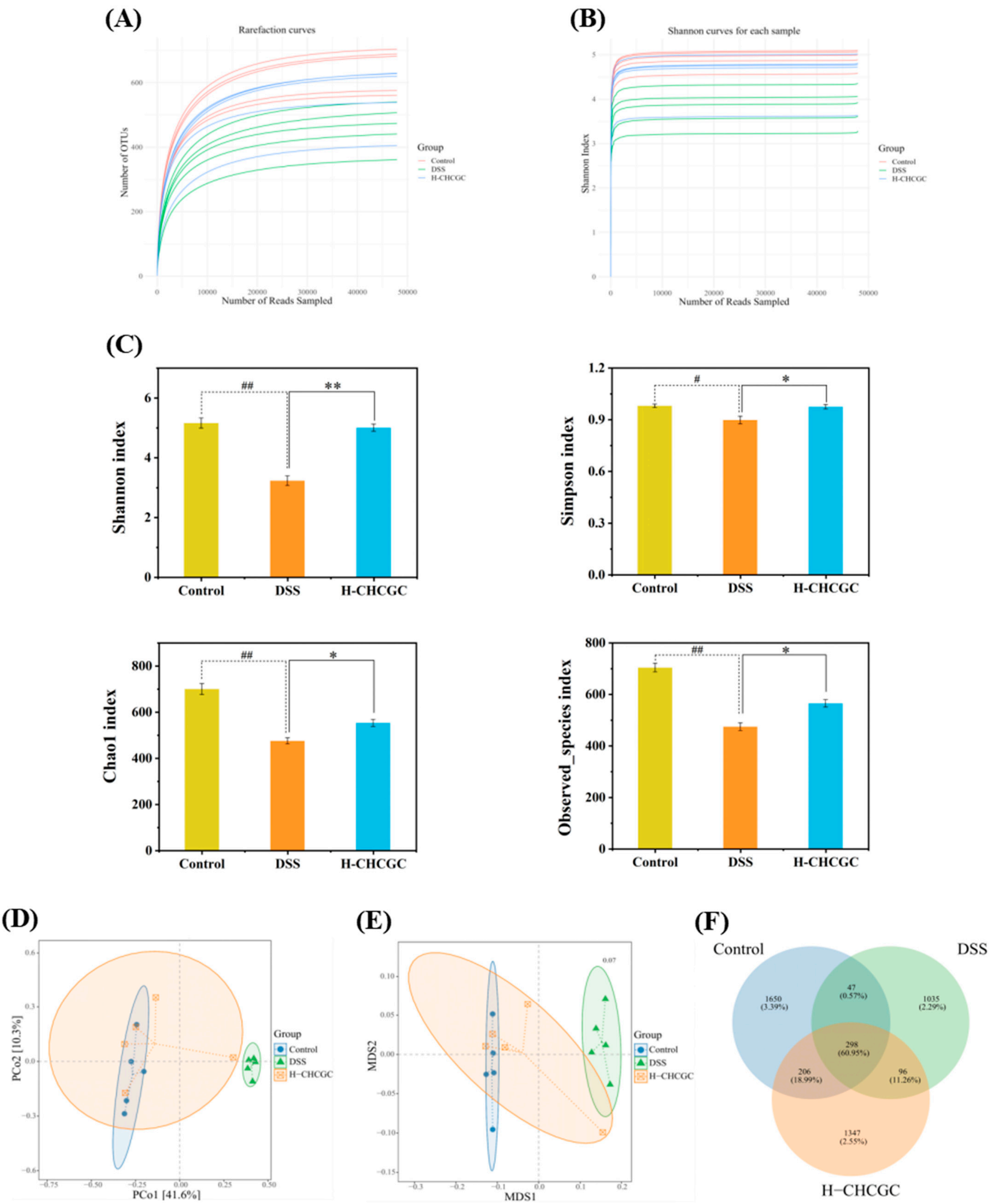

Supplementary Figure S4. High-dose CS/HHPC/Col I-GP-CurNPs (H-CHCGC) modulated DSS-induced gut microbiota dysbiosis in UC mice. (A) Rarefaction curves and (B) Shannon curves for different treatment groups. (C) α-diversity of the gut microbiota following different treatments. (D) PCoA and (E) NMDS plots showing β-diversity of the gut microbiota. (F) Venn diagram showing shared and unique species at the OTU level. Significance levels: $^{\#}p < 0.05$ and $^{\#\#}p < 0.01$ vs. control group; $^{*}p < 0.05$ and $^{**}p < 0.01$ vs. LPS group.

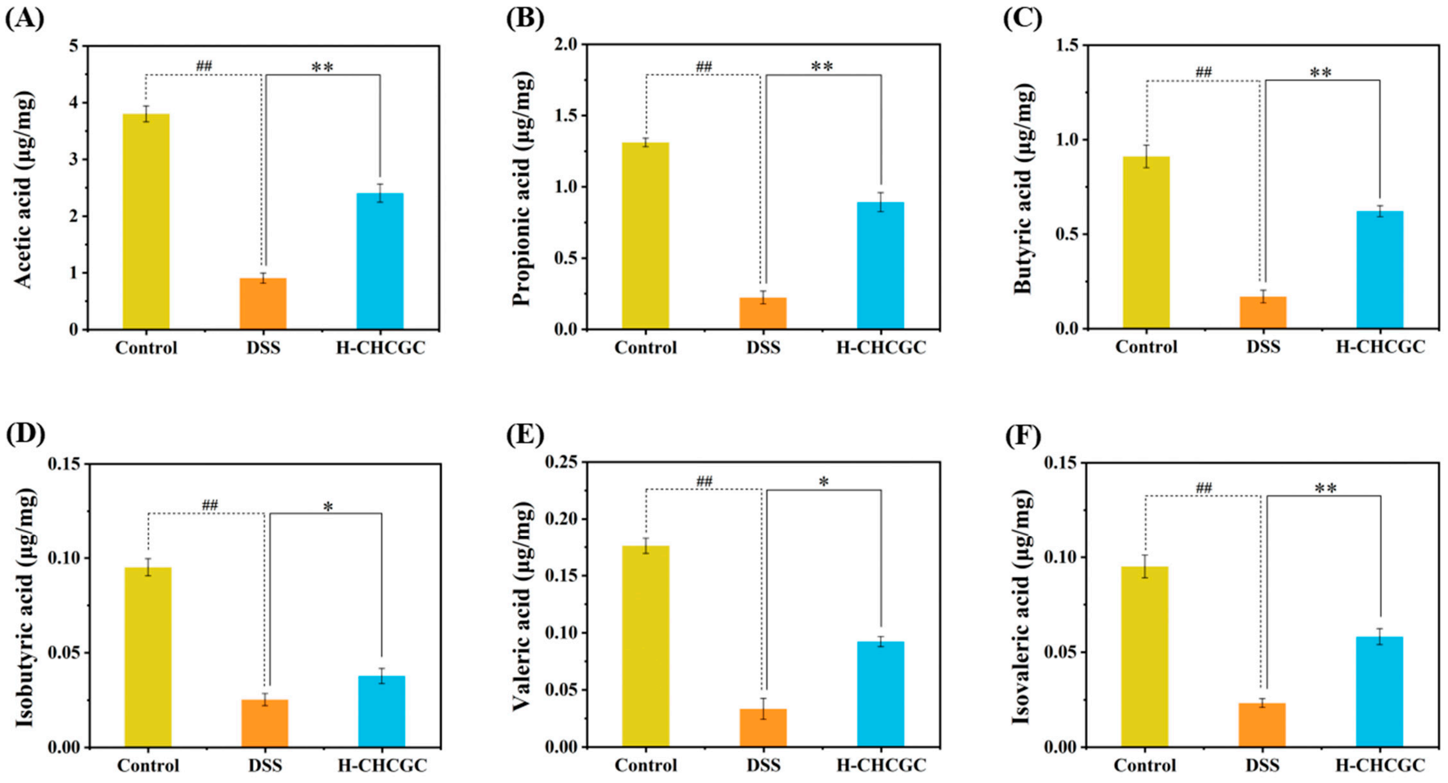

Supplementary Figure S5. SCFAs metabolite levels in cecal contents were determined by gas chromatography following different treatments. (A) Acetic acid, (B) propionic acid, (C) butyric acid, (D) isobutyric acid, (E) valeric acid, and (F) isovaleric acid. Significance levels: $^{\#}p$ < 0.05 and $^{\#\#}p$ < 0.01 vs. control group; *p < 0.05 and **p < 0.01 vs. LPS group.
